# Recognition of the True and False Resonance Raman Optical Activity

**DOI:** 10.1002/anie.202107600

**Published:** 2021-08-21

**Authors:** Ewa Machalska, Grzegorz Zajac, Aleksandra J. Wierzba, Josef Kapitán, Tadeusz Andruniów, Maciej Spiegel, Dorota Gryko, Petr Bouř, Malgorzata Baranska

**Affiliations:** ^1^ Faculty of Chemistry Jagiellonian University Gronostajowa 2 30-387 Krakow Poland; ^2^ Jagiellonian Centre for Experimental Therapeutics (JCET) Jagiellonian University Bobrzynskiego 14 30-348 Krakow Poland; ^3^ Institute of Organic Chemistry and Biochemistry Academy of Sciences Flemingovo náměstí 2 16610 Prague Czech Republic; ^4^ Institute of Organic Chemistry Polish Academy of Sciences Kasprzaka 44/52 01-224 Warsaw Poland; ^5^ Department of Optics Palacký University Olomouc 17. listopadu 12 77146 Olomouc Czech Republic; ^6^ Department of Chemistry Wroclaw University of Science and Technology Wyb. Wyspianskiego 27 50-370 Wroclaw Poland; ^7^ Department of Pharmacognosy and Herbal Medicine Wroclaw Medical University Borowska 211A 50-556 Wroclaw Poland

**Keywords:** chirality, electronic circular dichroism, Raman scattering, resonance Raman optical activity, vitamin B_12_

## Abstract

Resonance Raman optical activity (RROA) possesses all aspects of a sensitive tool for molecular detection, but its measurement remains challenging. We demonstrate that reliable recording of RROA of chiral colorful compounds is possible, but only after considering the effect of the electronic circular dichroism (ECD) on the ROA spectra induced by the dissolved chiral compound. We show RROA for a number of model vitamin B_12_ derivatives that are chemically similar but exhibit distinctively different spectroscopic behavior. The ECD/ROA effect is proportional to the concentration and dependent on the optical pathlength of the light propagating through the sample. It can severely alter relative band intensities and signs in the natural RROA spectra. The spectra analyses are supported by computational modeling based on density functional theory. Neglecting the ECD effect during ROA measurement can lead to misinterpretation of the recorded spectra and erroneous conclusions about the molecular structure.

## Graphical abstract

A strong influence of electronic circular dichroism on the measurement of resonance Raman optical activity of colorful chiral compounds is reported for a series of vitamin B_12_ derivatives. Neglecting this effect can lead to misinterpretation of recorded spectra, and seriously erroneous conclusions about molecular structure and chirality.

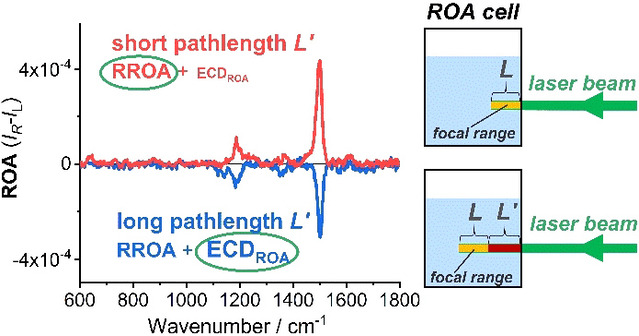

Chiroptical spectroscopy offers detailed insight into molecular handedness,^[1]^ which is particularly useful in the natural product characterization and detection.[^2^, ^3^] It also allows for monitoring the conformation or other morphological characteristics of biopolymers.^[4]^ Most common chiroptical methods include vibrational and electronic circular dichroism (VCD, ECD), arising from different absorption of the left‐ and right‐circularly polarized light (CPL), and Raman optical activity (ROA), which measures the intensity difference in Raman scattering of right‐ and left‐CPL.^[5]^ For natural compounds, ECD spectroscopy enables to study small changes in the electronic structure, as demonstrated successfully on a set of corrinoid compounds differing in the upper axial ligand,^[6]^ while ROA method has found a number of applications in cancer diagnostics^[7]^ and biomolecules sensing.^[8]^

Recently, several developments have been made in the experimental[^9^, ^10^, ^11^, ^12^, ^13^, ^14^] and computational[^15^, ^16^, ^17^, ^18^] methodologies of the pre‐resonance and resonance ROA (RROA). The effort is stimulated by the weakness of the “classical” non‐resonance ROA scattering. We estimate that one photon in a million passing through a typical sample is Raman‐scattered in our experiments. ROA is typically four orders of magnitude weaker than Raman scattering, and due to statistical nature of photon detection (shot noise) we need to record ≈10^10^ (!) such events in order to detect ROA with reasonable (10:1) signal to noise ratio. Under resonance when the energy of incident laser radiation matches energy of an electronic transition the efficiencies of Raman scattering and ROA measurement significantly increase. RROA can be detected at low concentrations (ca. 10^−5^ M)[^19^, ^20^, ^21^, ^22^] while maintaining structural sensitivity.[^12^, ^21^, ^23^] Single state resonance usually leads to single signed RROA,[^1^, ^19^, ^24^] whereas pre‐resonance and more resonance states provide RROA spectrum with positive and negative bands, similarly as for the “conventional” non‐resonance case. However, up to now there is a limited number of reports on RROA, due to the challenges arising during the measurement, including artifacts related to absorption and fluorescence.[^12^, ^13^, ^14^, ^21^, ^23^, ^25^]

RROA is associated with an interesting phenomenon of “chirality transfer” which sheds new light on molecular interactions. A chirality induction/transfer can be observed either when achiral molecules are in a close proximity to the chiral ones^[19]^ or due to a plasmonic resonance.^[8]^ A strong induced optical activity under resonance conditions was reported to occur from chiral metal complexes^[26]^ or dyes[^22^, ^27^, ^28^] to numerous (a)chiral organic solvents. The phenomenon was initially explained by a phenomenological model named “ring of fire”,^[26]^ partially predicting the spectra, in particular signs of the ROA bands. More recently, an interference between the ROA and ECD better explained the effect, including prediction of band intensities.^[27]^ The importance of ECD became evident in double‐cell experiments, where there was no chemical contact between the solute and the solvent.

Intrigued by the ECD/ROA interference, we envisaged that it would be significant not only for the *solvent* spectra but also for chiral, light‐absorbing *solutes*. Indeed, in the present study, we show that the measured ROA of chiral colorful compounds is a sum of the true RROA and the ECD‐induced parts (ROA_ECD_):

(1) $$\mathrm{RROA}\;=\;\mathrm{RROA}{}_{\mathrm{true}}\;+\;\mathrm{ROA}{}_{\mathrm{ECD}}$$

Since the ROA_ECD_/Raman ratio is dependent on the concentration, the same holds for measured RROA. Because ROA_ECD_ can be more or less accurately simulated, also the true molecular part (RROA_true_) can be extracted from the experimental spectra. This is explored for a series of chiral organometallic vitamin B_12_ analogues (Figure 1),[^29^, ^30^, ^31^] which strongly scatter light. Their fluorescence, which would otherwise compete with ROA measurement, is reasonably weak. The analogues differ in structure around the main chromophore. The different substitutions on the corrin ring lead to large variations in the intensity and shape of UV/Vis and ECD absorption bands, and they also determine the behavior in ROA experiments.

**Figure 1:**
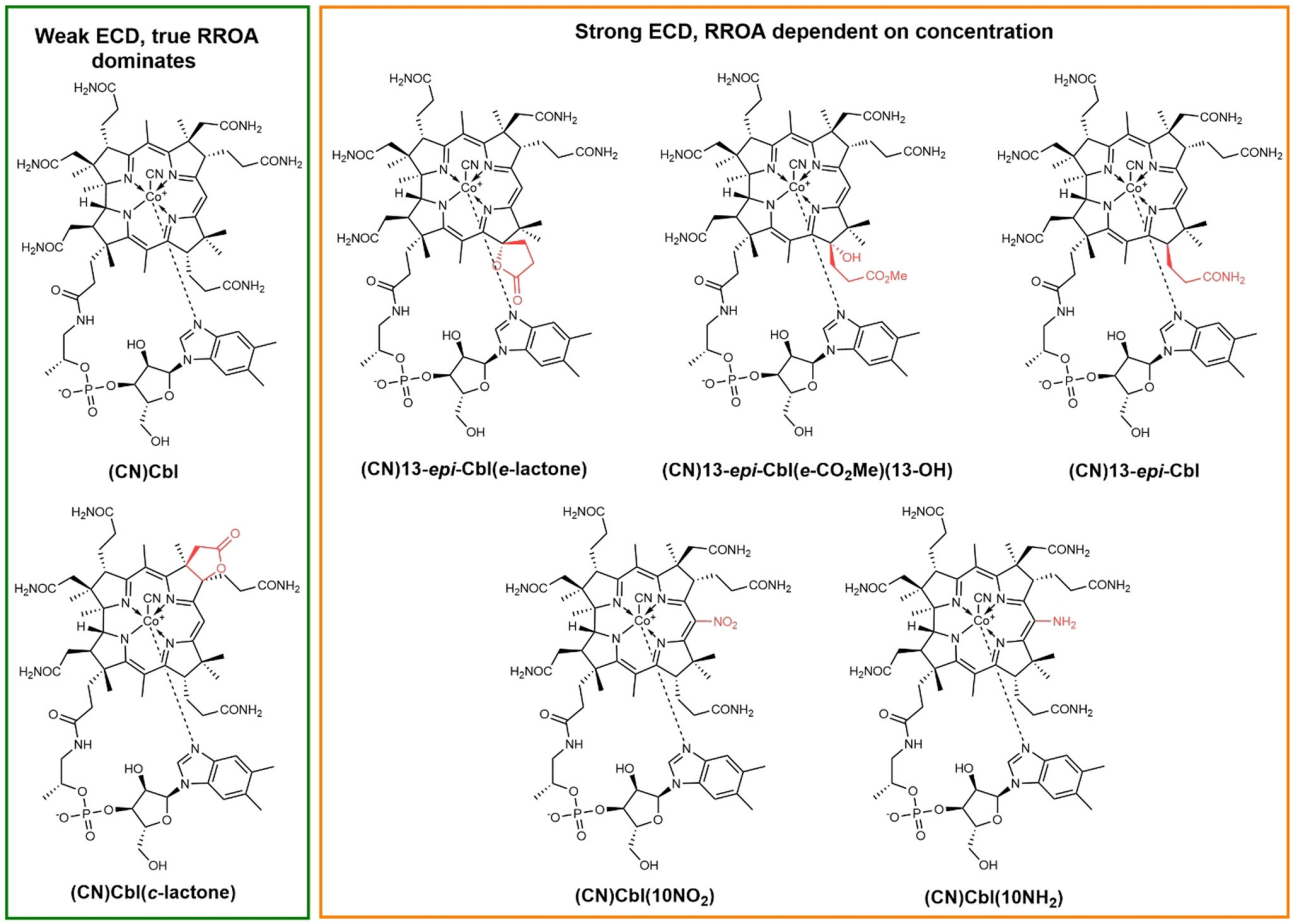
Structures of studied vitamin B_12_ analogues, divided into two groups according to their behavior in RROA experiments. Variations from the native vitamin B_12_ structure are marked in red.

Vitamin B_12_ (cobalamin) is a chiral organometallic molecule (Figure 1) possessing a number of functional groups available for chemical modifications, thereby it is possible to combine its native structure with various biologically active molecules.^[32]^ Consequently, vitamin B_12_ was applied as a delivery tool into bacterial^[33]^ or mammalian^[34]^ cells and used to alter the bioavailability of proteins^[35]^ or anti‐cancer drugs.^[36]^ Cobalamins exhibit all relatively strong ECD close to the excitation laser wavelength (532 nm) and therefore may induce not only ROA_ECD_ signals in the solvent, but also in their own vibrational bands. We believe that this phenomenon has been either overlooked or misinterpreted in earlier studies.

The ROA_ECD_ interference is due to different absorption of the incident and scattered left and right circularly polarized light, as it passes through the sample solution. For the ratio of ROA and Raman intensities (*CID*—circular intensity difference), a simplified equation has been proposed.^[27]^ We use a slightly modified [Equation 2],

(2) $$CID=\frac{I_{R}-I_{L}}{I_{R}+I_{L}}≅f\frac{\Delta \epsilon {}^{'}+DOC\Delta \epsilon }{4}cLln\left(10\right)$$

where *I_R_* and *I_L_* are intensities of right‐ and left‐CPL detected by the ROA/Raman spectrometer; Δ*ϵ* and Δ*ϵ*′ are decadic absorption coefficients (ECD intensities) at the incident and scattered light frequency, respectively, *DOC* is the degree of circularity characteristic for each vibration, and *c* is concentration of the compound that exhibits the ROA_ECD_ effect. In the correction factor, $f=1+\frac{2L^{'}}{L}$ , *L*′ is the part of optical path length not involved in the scattering, and *L* is a Raman active path length (length of the focal range, cf. Figure S6). We used *L*=2 mm and *L*′=1 mm. *DOC* can be related to the Raman depolarization ratio, and varies from +5/7 for depolarized bands to −1 for completely polarized bands, in the conditions far from the resonance.^[1]^ As it was described recently,^[27]^ there are several limit cases according to ECD intensities at the incident and the scattered light wavelengths. If Δ*ϵ*′≈Δ*ϵ*≈const. (ECD signal is constant in the scattering ROA range), ROA_ECD_ is monosigned, and the sign is the same as for ECD. For |Δ*ϵ*′|<|Δϵ| the *DOC*Δ*ϵ* product may dominate, and the ROA_ECD_ signal may be in general both positive and negative, according to the *DOC* values. Similarly for |Δ*ϵ*′|>|Δ*ϵ*| ROA_ECD_ follows the ECD sign in the scattering range.

By studying vitamin B_12_ analogues, we discovered that the level of ROA_ECD_ interference strongly depends on the ECD of the studied chiral compound, its concentration, and the pathlength of the light propagation through the sample. This interference can be easily observed for molecules with a relatively high ECD signal in the range of ROA scattering (0–2500 cm^−1^, which corresponds to 532–610 nm).

We have found that some vitamin B_12_ derivatives exhibit a concentration dependence of the RROA signal, showing even RROA signal sign flipping. The pronounced dependence on the concentration is observed for the analogues with pronounced Δ*ϵ* values, (CN)13‐*epi*‐Cbl(*e*‐lactone), (CN)13‐*epi*‐Cbl, (CN)13‐*epi*‐Cbl(*e*‐CO_2_Me)(13‐OH), and (CN)Cbl(10‐NO_2_). In contrast, nearly independent of the concentration are RROA spectra of native (CN)Cbl and (CN)Cbl(*c*‐lactone) which exhibit small ECD.

The behavior of (CN)13‐*epi*‐Cbl(*e*‐lactone) and (CN)Cbl is documented in Figure 2, other examples are given in ESI. For (CN)Cbl, only slight changes in RROA intensities are observed, which is also reflected in the weak dependence of CIDs (1500 cm^−1^ band, mainly C=C corrin ring stretching)^[21]^ on the concentration (Figure 2). Note that the Raman and RROA spectra were normalized to maximum in the Raman spectrum, but the CID values (ROA/Raman ratios) are preserved. A more pronounced dependence is observed for the (CN)13‐*epi*‐Cbl(*e*‐lactone), where RROA and CID are positive for the two lowest concentrations (0.1 and 0.2 mg mL^−1^), RROA is bisignate and CID of 1500 cm^−1^ is close to zero for 0.4 mg mL^−1^, and for the three highest concentrations (0.6, 0.8 and 1.0 mg mL^−1^) RROA and CIDs are negative.

**Figure 2:**
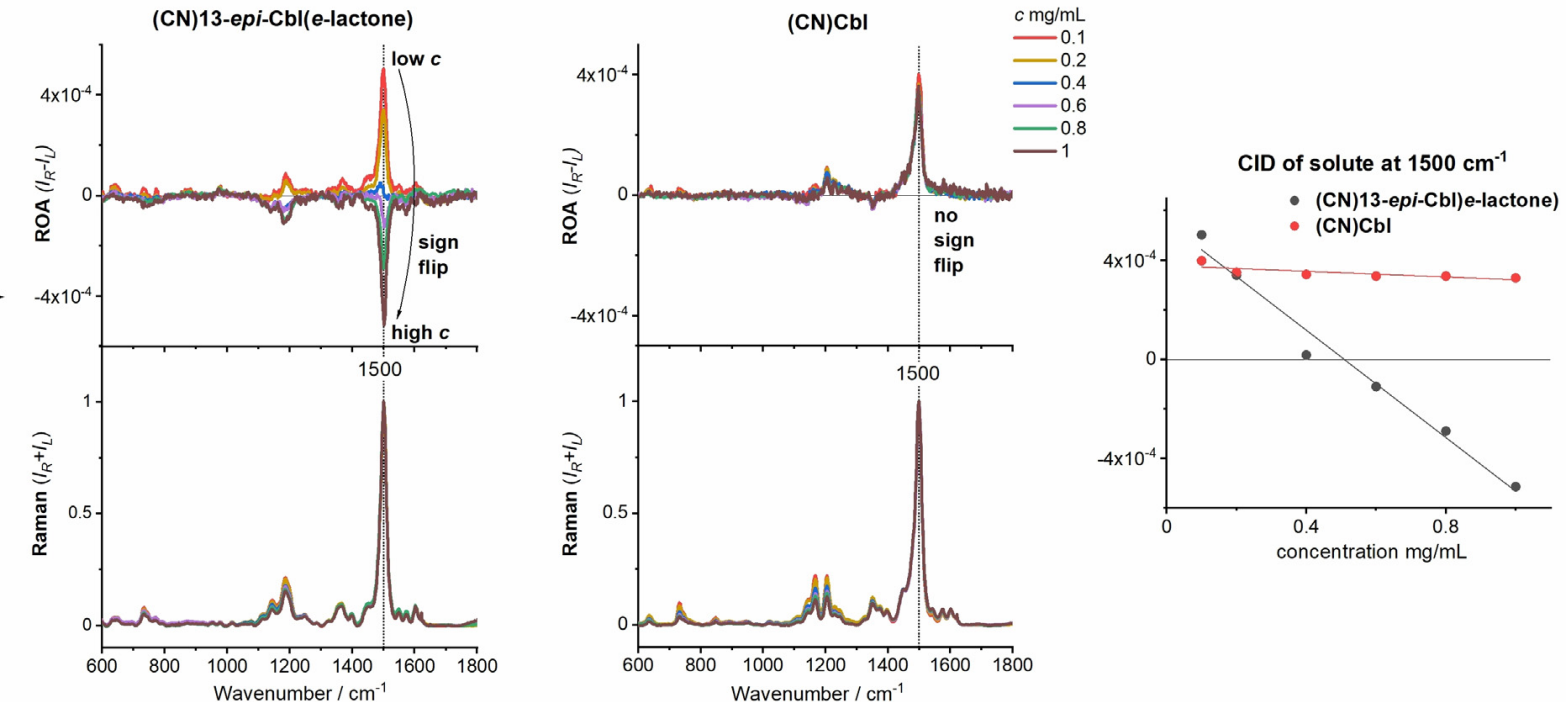
ROA and Raman spectra, and CID values measured for various concentrations of (CN)13‐*epi*‐Cbl(*e*‐lactone) and (CN)Cbl in water. The spectra are normalized to the strongest Raman band.

We originally considered that at higher concentrations the chemical structure of the studied compound might change due to e.g., laser‐induced decomposition. However, ECD/UV/Vis spectra of all studied compounds, measured before and after the ROA measurements remained unchanged (ESI). Also a dilution of previously measured (CN)13‐*epi*‐Cbl(*e*‐lactone) solutions, from 0.8 mg mL^−1^ to 0.1 mg mL^−1^, gave the same ROA as freshly prepared 0.1 mg mL^−1^ solution (ESI). Therefore, we concluded that the observed concentration dependence can be attributed to the ROA_ECD_ interference.

The presence of ROA_ECD_ is also indicated by ECD of (CN)13‐*epi*‐Cbl(*e*‐lactone) in the scattering region, which is much higher in the absolute value than for (CN)Cbl (Figure S12, ESI). ECD of (CN)13‐*epi*‐Cbl(*e*‐lactone) is negative and approximately constant in the range of the most prominent ROA bands (532–581 nm–0–1600 cm^−1^), for which [Equation (2)] gives monosigned negative ROA_ECD_. This is consistent with the experiment and simulated spectra discussed below.

The two compounds provide different ECD spectra because of the propionate side chain is projected up instead of down to the plain of the corrin ring, and the presence of the spiro‐lactone group at this position. This affects the corrin ring electronic structure and leads to the slight difference in the nature of the corrin ring electronic transitions (ESI). Because the observed RROA signal is a sum of molecular RROA_true_ and ROA_ECD_ contribution, the lower the concentration, the closer we are to the pure (true) RROA effect. Due to the low ECD of (CN)Cbl in the ROA scattering spot, the ROA_ECD_ here does not contribute significantly.

Another way to minimize ROA_ECD_ interference is to shift the focal range closer to the cell wall. The focal range is given by instrumental optics transferring the scattered radiation towards the spectrograph. When it is in the middle of the cell (*L*′ is comparable to *L*), the contribution of the interference is more pronounced or even dominant. Indeed, this was observed for (CN)13‐*epi*‐Cbl(*e*‐lactone) (Figure 3).

**Figure 3:**
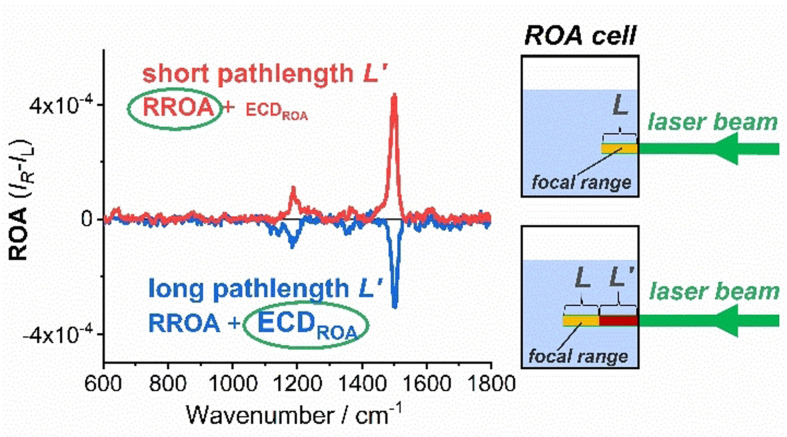
ROA spectra of (CN)13‐*epi*‐Cbl(*e*‐lactone) at 0.8 mg mL^−1^ concentration measured for different cell positions: focal range of length *L* located close to cell wall (RROA dominates) and focal range located at distance *L*′ from the wall (ECD_ROA_ dominates).

Similarly to previously described compounds,^[27]^ cobalamins can induce ROA in the solvent. Contrary to water solutions, Raman spectrum of (CN)Cbl in DMSO is dominated by the solvent signal and a strong ROA_ECD_ interference (Figure S24, S25, ESI). Both (CN)Cbl and (CN)13‐*epi*‐Cbl(*e*‐lactone) give negative ROA_ECD_ solvent ROA bands, due to negative ECD in the scattering range. The effect is much more pronounced for (CN)13‐*epi*‐Cbl(*e*‐lactone), due to its bigger ECD. An increased ROA_ECD_ interference in the case of (CN)13‐*epi*‐Cbl(*e*‐lactone) is observed both for vibrational bands of the compound and DMSO. Similarly as for water solutions the registered ROA signal of (CN)13‐*epi*‐Cbl(*e*‐lactone) in DMSO is linearly dependent on the concentration of the solute, and becomes negative at higher concentrations. ROA_ECD_ bands of DMSO are always negative and linearly dependent on a concentration. Worth mentioning is that for both compounds the ECD intensity at (532–610 nm) is higher for DMSO solutions compared to water (Figure S12, ESI). The solvent dependence is especially strong for (CN)Cbl where ECD signal changes from about −1 dm^3^⋅mol^−1^⋅cm^−1^ for water to about ‐5 dm^3^⋅mol^−1^⋅cm^−1^ for DMSO. This causes a substantial increase of the ROA_ECD_ interference observed in ROA spectra of the compound itself and of the solvent.

In the modelling the solvent dependence is difficult to account for, as the bulk simulations of spectroscopic properties are quite time‐demanding.^[37]^ On the other hand, our simulations of B_12_ ECD and ROA spectra conducted in vacuum and with PCM solvent model provided qualitatively similar results, indicating that the solute‐solvent interactions do not significantly change the properties of the inner metal/corrin core.

In accord with the experiment, simulations based on [Equation (2)] predict almost no ROA_ECD_ for (CN)Cbl, while for (CN)13‐*epi*‐Cbl(*e*‐lactone) the bands are negative, relatively strong, and linearly dependent on the concentration (Figure 4, Figure S7, ESI). Also simulations of ROA_ECD_ of DMSO are consistent with the experimental results (ESI).

**Figure 4:**
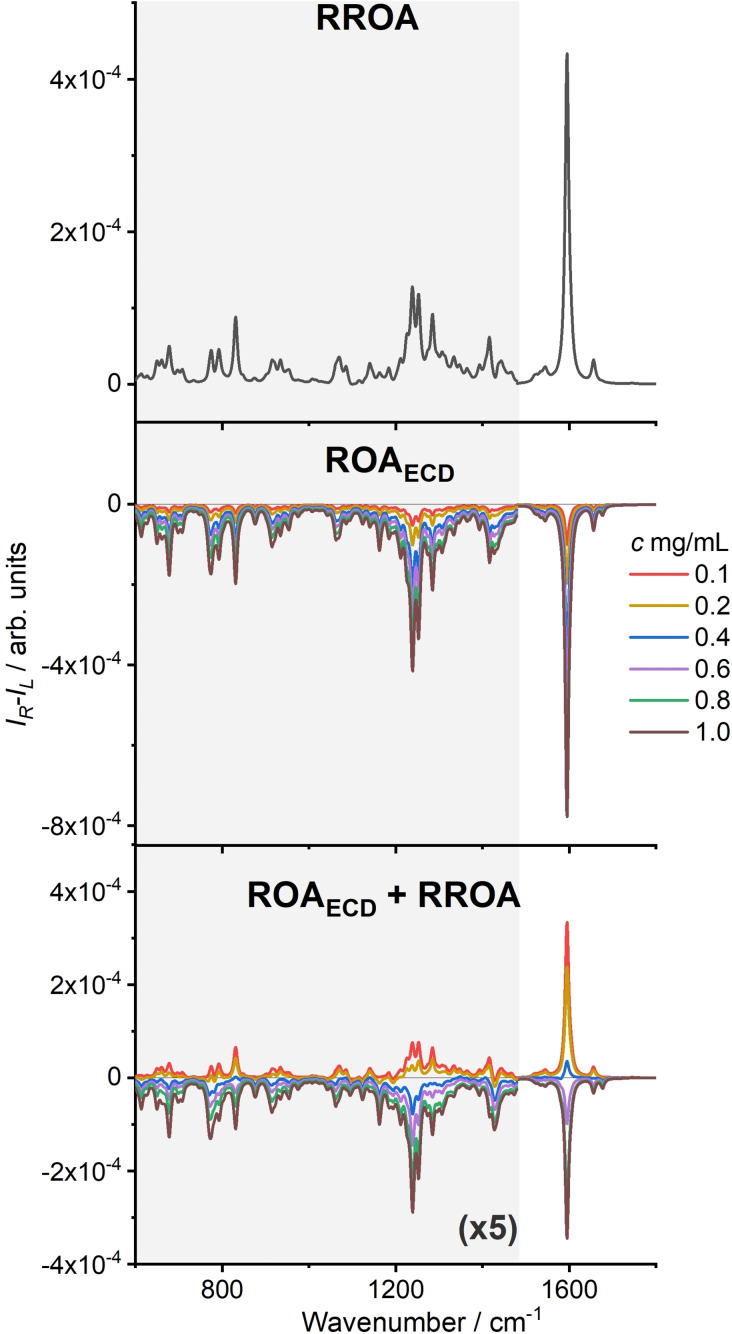
Simulated ROA spectra of (CN)13‐*epi*‐Cbl(*e*‐lactone), the RROA, and ROA_ECD_ components and ROA_ECD_ + RROA sum as dependent on the concentration, for L=2 mm and L′=1 mm in [Equation (2)].

An increasing number of reports on the use of ROA of absorbing molecules proves the importance of this subject for the physicochemical community. In the reviewing process of this paper an interesting question how much the ECD effect affects circular polarized luminescence (CPL) appeared. CPL is often recorded during ROA experiments,[^38^, ^39^] however, it is often stronger and the role of the ECD influence on it needs to be examined in future studies.

Herein, we investigated the influence of the ECD on the RROA or pre‐RROA spectra and showed that neglecting this phenomenon may lead to misinterpretation and wrong conclusions about sample properties. Fortunately, the interaction of RROA and ROA_ECD_ can be reasonably well estimated from experimental molar extinction coefficient, molar circular dichroism and circularly polarized Raman spectra. The RROA_true_ component can thus be extracted from the experimental data. This was shown here for model vitamin B_12_ analogues, but the procedure is general for other absorbing chiral compounds. We are convinced that the presented results will be of interest particularly to the spectroscopic community as they will allow us for a better control of the experimental conditions and lead to the proper interpretation of the experiment.

## Conflict of interest

The authors declare no conflict of interest.

## Supporting information

As a service to our authors and readers, this journal provides supporting information supplied by the authors. Such materials are peer reviewed and may be re‐organized for online delivery, but are not copy‐edited or typeset. Technical support issues arising from supporting information (other than missing files) should be addressed to the authors.

Supporting Information
