# Supplementary material for: Recognition of the True and False Resonance Raman Optical Activity
Source: Angew Chem Int Ed Engl. 2021 Aug 21;60(39):21205–10. doi: 10.1002/anie.202107600 (PMC8519086; doi:10.1002/anie.202107600)
Supplement: Supplementary file 1 — Supporting Information [file ANIE-60-21205-s001.pdf]

Angewandte Chemie
Eine Zeitschrift der Gesellschaft Deutscher Chemiker

Supporting Information

Recognition of the True and False Resonance Raman Optical Activity

Ewa Machalska[+], Grzegorz Zajac[+], Aleksandra J. Wierzba, Josef Kapitán, Tadeusz Andruniów, Maciej Spiegel, Dorota Gryko,* Petr Bouř,* and Malgorzata Baranska**

anie_202107600_sm_miscellaneous_information.pdf

Contents

Calculations 3

Table S1. Standard Gibbs free energies and Boltzmann populations of the most stable conformers of (CN)13-*epi*-Cbl(*e*-lactone) 3

Table S2. Standard Gibbs free energies and Boltzmann populations of the most stable conformers of (CN)Cbl 3

Figure S1. Comparison of the optimized structures of the most stable conformers of (CN)13-*epi*-Cbl(*e*-lactone) and (CN)Cbl 4

Figure S2. Comparison of experimental and calculated UV-Vis/ECD spectra of (CN)13-*epi*-Cbl(*e*-lactone) and (CN)Cbl 5

Table S3. Calculated energies, oscillator and rotatory strengths of the electronic transitions of the lowest conformers of (CN)Cbl and (CN)13-*epi*-Cbl(*e*-lactone) 6

Table S4. Dominant molecular orbital excitations and energies, oscillator and rotatory strengths of the first three electronic transitions of (CN)Cbl and (CN)13-*epi*-Cbl(*e*-lactone) 6

Figure S3. Molecular orbitals dominant in the first 3 electronic states of (CN)13-*epi*-Cbl(*e*-lactone) and (CN)Cbl 7

Figure S4. Molecular orbitals dominant in the first 3 electronic states of (CN)13-*epi*-Cbl(*e*-lactone) and (CN)Cbl (continuation) 8

Figure S5. Transition charge densities for the first 3 electronic transitions of (CN)13-*epi*-Cbl(*e*-lactone) and (CN)Cbl 9

ROA_{ECD} calculations 10

Figure S6. Laser focusing scheme in the ROA experiment 10

Figure S7. Theoretical ROA spectra of (CN)Cbl: *pre*-RROA, ROA_{ECD} simulation, sum of ROA_{ECD} + *pre*-RROA spectra 10

Figure S8. Experimental ROA spectra of (CN)Cbl and (CN)13-*epi*-Cbl(*e*-lactone) compared with calculated ROA_{ECD} signal of DMSO 11

Figure S9. ROA_{ECD} signal of DMSO spectra for (CN)13-*epi*-Cbl(*e*-lactone) and (CN)Cbl in DMSO 11

Experimental Procedures 12

Table S5. Set of measurement parameters of studied compounds dissolved in water or DMSO 13

Figure S10. UV-Vis/ECD spectra of vitamin B_{12} and its analogues in water recorded before and after ROA experiments 14

Figure S11. UV-Vis/ECD spectra of vitamin B_{12} and (CN)13-*epi*-Cbl(*e*-lactone) in DMSO recorded before and after ROA experiments 15

Figure S12. Comparison ECD spectra of (CN)Cbl and (CN)13-*epi*-Cbl(*e*-lactone) in water and DMSO 15

Figure S13. Raw Raman/ROA spectra of vitamin B_{12} in DMSO at different concentrations 16

Figure S14. Raw Raman/ROA spectra of (CN)13-*epi*-Cbl(*e*-lactone) in DMSO at different concentrations 17

Figure S15. Raw Raman/ROA spectra of vitamin B_{12} in water at different concentrations. 18

Figure S16. Raw Raman/ROA spectra of (CN)13-*epi*-Cbl(*e*-lactone) in water at different concentrations 19

Figure S17. Raw Raman/ROA spectra of (CN)13-*epi*-Cbl in water at different concentrations. 20

Figure S18. Raw Raman/ROA spectra of (CN)13-*epi*-Cbl(*e*-CO_2Me)(13-OH) in water at different concentrations 21

Figure S19. Raw Raman/ROA spectra of (CN)Cbl(10-NO_2) in water at different concentrations. 22

Figure S20. Raw Raman/ROA spectra of (CN)Cbl(10-NH_2) in water at different concentrations 23

Figure S21. Raw Raman/ROA spectra of (CN)Cbl(*c*-lactone) in water at different concentrations 23

Figure S22. Raw Raman/ROA spectra of (CN)13-*epi*-Cbl(*e*-lactone) in water registered with different path lengths. 24

Figure S23. Raw Raman/ROA spectra of (CN)13-*epi*-Cbl(*e*-lactone) in water diluted from 0.8 to 0.1 mg/mL concentration 25

Figure S24. Normalized ROA/Raman spectra of (CN)Cbl and (CN)13-*epi*-Cbl(*e*-lactone) in DMSO and comparison of CID values 26

Figure S25. ROA/Raman spectra of (CN)13-*epi*-Cbl(*e*-lactone) and (CN)Cbl in DMSO 26

Organic synthesis 27

Figure S26. ^{1}H NMR spectrum of (CN)13-*epi*-Cbl 27

Figure S27. ^{13}C NMR spectrum of (CN)13-*epi*-Cbl 28

References 28

Calculations

MD simulations

(CN)13-*epi*-Cbl(*e*-lactone) and (CN)Cbl initial geometries have been built in Avogadro software.[1] The molecular dynamics (MD) conformational search tool implemented in Gabedit software[2] was recruited to generate 10 lowest-energy conformers of the studied molecular systems. Amber99 force field[3] was employed. Simulated annealing procedure (T=1000 K) included heating, equilibration and production runs which lasted 1, 1, and 10 ps, respectively. The time step was 1 fs. Velocity Verlet algorithm was employed in trajectory calculations. No constraints were used. At the end of the molecular dynamics calculations the selected lowest energy conformers were optimized using quasi Newton Raphson procedure (10 000 steps). The optimization convergence criteria are the default ones as defined in Gabedit software.

Quantum chemical calculations

All quantum chemical calculations were performed by of Gaussian G16.C01 software,[4] and analyzed by means of GaussView 6,[5] VMD 1.9.4,[6] GaussSum 3 software,[7] and a set of home-made programs and scripts.
A set of conformers of (CN)13-*epi*-Cbl(*e*-lactone) and (CN)Cbl obtained from the MD simulations was optimized at the CAM-B3LYP/GD3/6-31G(d) theory level. The MDF10 pseudopotential and basis set were used for the Co atom. The solvent (water) was modeled using the CPCM model.[8]
Electronic absorption energies and intensities (oscillator and rotatory strengths) were calculated using TD-DFT, for the first 100 electronic states, at the same level of theory as for the geometries. Similarly, vibrational frequencies and pre-resonance Raman and ROA intensities were calculated employing the same level for the lowest-energy conformers of (CN)13-*epi*-Cbl(*e*-lactone) and (CN)Cbl. The excitation wavelength used in the polarizability calculation was close to the first electronic transitions, to mimic the experimental conditions. In particular, although the incident laser wavelength is 532 nm, theoretical electronic transition energies are blue-shifted compared to the experiment, and the excitation wavelength was adapted accordingly, the value of 430 nm provided the best results. Smooth theoretical UV-Vis/ECD and Raman/ROA spectra were obtained by a convolution with Gaussian functions of 0.1 eV and Lorentzian functions of 10 cm^{-1} half width at half maximum, respectively.

Table S1. Standard Gibbs free energies and Boltzmann populations (at 25°C) of the most stable conformers of (CN)13-*epi*-Cbl(*e*-lactone), obtained from MD simulations in H_2O.

Conformer	ΔG / hartree	Relative ΔG / kJ mol^{-1}	Population / %
1	-4757.612577	0.00	89.9
2	-4757.609951	6.89	5.6
3	-4757.609329	8.53	2.9
4	-4757.608662	10.28	1.4

Table S2. Standard Gibbs free energies and Boltzmann populations (at 25°C) of the most stable conformers of (CN)Cbl, obtained from MD simulations
in H_2O.

Conformer	ΔG / hartree	Relative ΔG / kJ mol^{-1}	Population / %
1	-4776.309977	0.00	85.2
2	-4776.308325	4.34	14.8

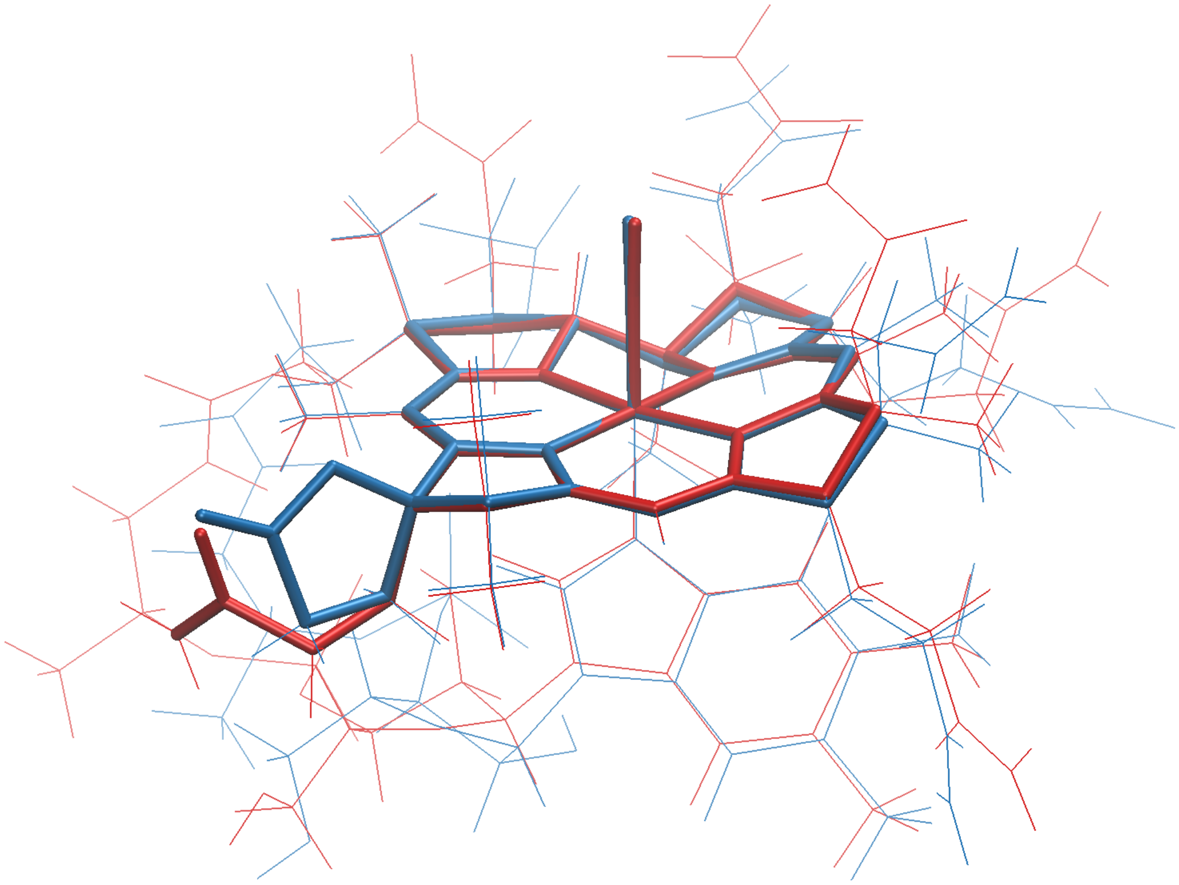

Figure S1. Comparison of the optimized structures of the most stable conformers of (CN)13-*epi*-Cbl(e-lactone) (blue) and (CN)Cbl (red). Corrin ring and *c*-side chains are bold.

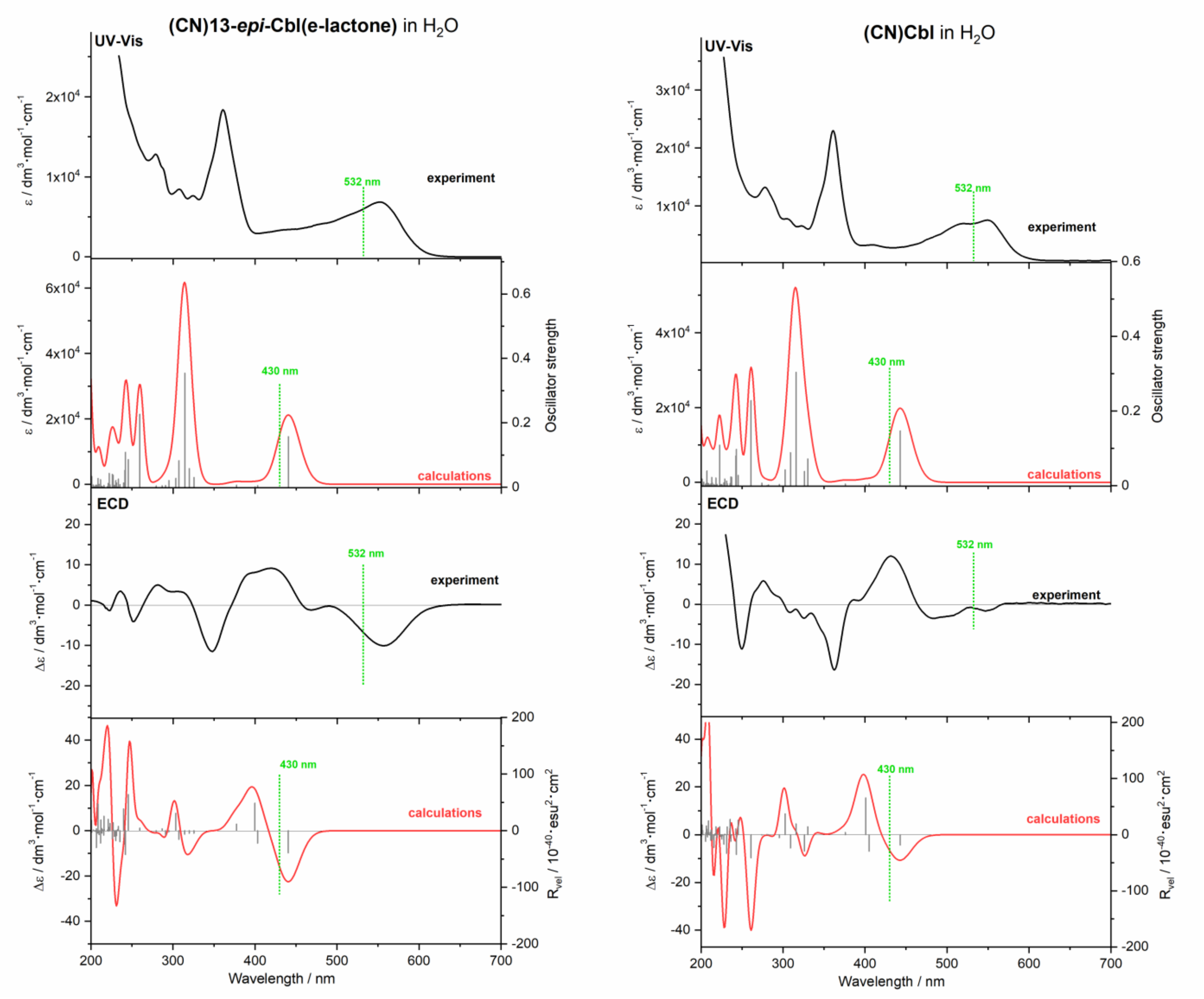

Figure S2. Comparison of experimental (black) and calculated (red) UV-Vis and ECD spectra of (CN)13-*epi*-Cbl(*e*-lactone) and (CN)Cbl. Individual transition energies and their oscillatory and rotatory strengths are indicated by the black vertical lines. The 532 nm excitation wavelength and that one used in the calculations are indicated by the green dotted lines.

Table S3. Calculated energies (in nm), oscillator (f) and rotatory (R_{vel}) strengths of the first 10 electronic transitions of the lowest-energy conformers of (CN)Cbl and (CN)13-*epi*-Cbl(*e*-lactone).

(CN)Cbl

state	λ / nm	f	R_{vel} / 10^{-40}·esu^2·cm^2
1	442.85	0.1469	-18.5998
2	404.89	0.0051	-29.4813
3	400.81	0.0020	65.6341
4	375.87	0.0046	4.0422
5	330.20	0.0718	14.2289
6	326.10	0.0385	-29.2861
7	315.85	0.3037	19.3908
8	309.05	0.0886	-23.6618
9	302.54	0.0427	37.0635
10	295.35	0.0035	-5.4094

(CN)13-*epi*-Cbl(*e*-lactone)

state	λ / nm	f	R_{vel} / 10^{-40}·esu^2·cm^2
1	440.56	0.1571	-39.0634
2	403.15	0.0050	-21.8730
3	399.87	0.0011	48.7356
4	377.45	0.0063	11.4726
5	325.48	0.0305	-4.2821
6	319.87	0.0579	-4.2794
7	314.33	0.3539	-5.9154
8	307.19	0.0824	-15.2125
9	303.36	0.0276	30.4427
10	294.94	0.0208	-1.8196

Table S4. Dominant molecular orbital (MO) excitations and energies (in nm), oscillator (f) and rotatory (R_{vel} strengths) of the first three electronic transitions of (CN)Cbl and (CN)13-*epi*-Cbl(*e*-lactone).

(CN)Cbl

state	λ / nm	f	R_{vel}	excitation	%	donor MO	acceptor MO
1	442.85	0.1469	-18.5998	354→355	91	cor-*π* (HOMO)	cor-*π** (LUMO)
2	404.89	0.0051	-29.4813	320→357	9	cor-*π*/CN-*π*/Co $3d_{xz}$ $3d_{yz}$	Co $3d_z{}^2$/cor-*π**/CN-*π**
				352→357	9	cor-*π*/DMB-*π*/ Co $3d_{xz}$ $3d_{yz}$	Co $3d_z{}^2$/cor-*π**/CN-*π**
3	400.81	0.0020	65.6341	326→357	17	cor-*π*/CN-*π*/Co $3d_{xz}$ $3d_{yz}$	Co $3d_z{}^2$/cor-π*/CN-*π**

(CN)13-*epi*-Cbl(*e*-lactone)

state	λ / nm	f	R_{vel}	excitation	%	donor MO	acceptor MO
1	440.56	0.1571	-39.0634	353→354	91	cor-*π* (HOMO)	cor-*π** (LUMO)
2	403.15	0.0050	-21.8730	350→356	17	cor-*π*/ Co $3d_{xz}$ $3d_{yz}$	Co $3d_z{}^2$/cor-*π**/CN-*π**
3	399.87	0.0011	48.7356	326→356	12	cor-*π*/ DMB-*π*/CN-*π*/Co $3d_{xz}$ $3d_{yz}$	Co $3d_z{}^2$/cor-π*/CN-*π**

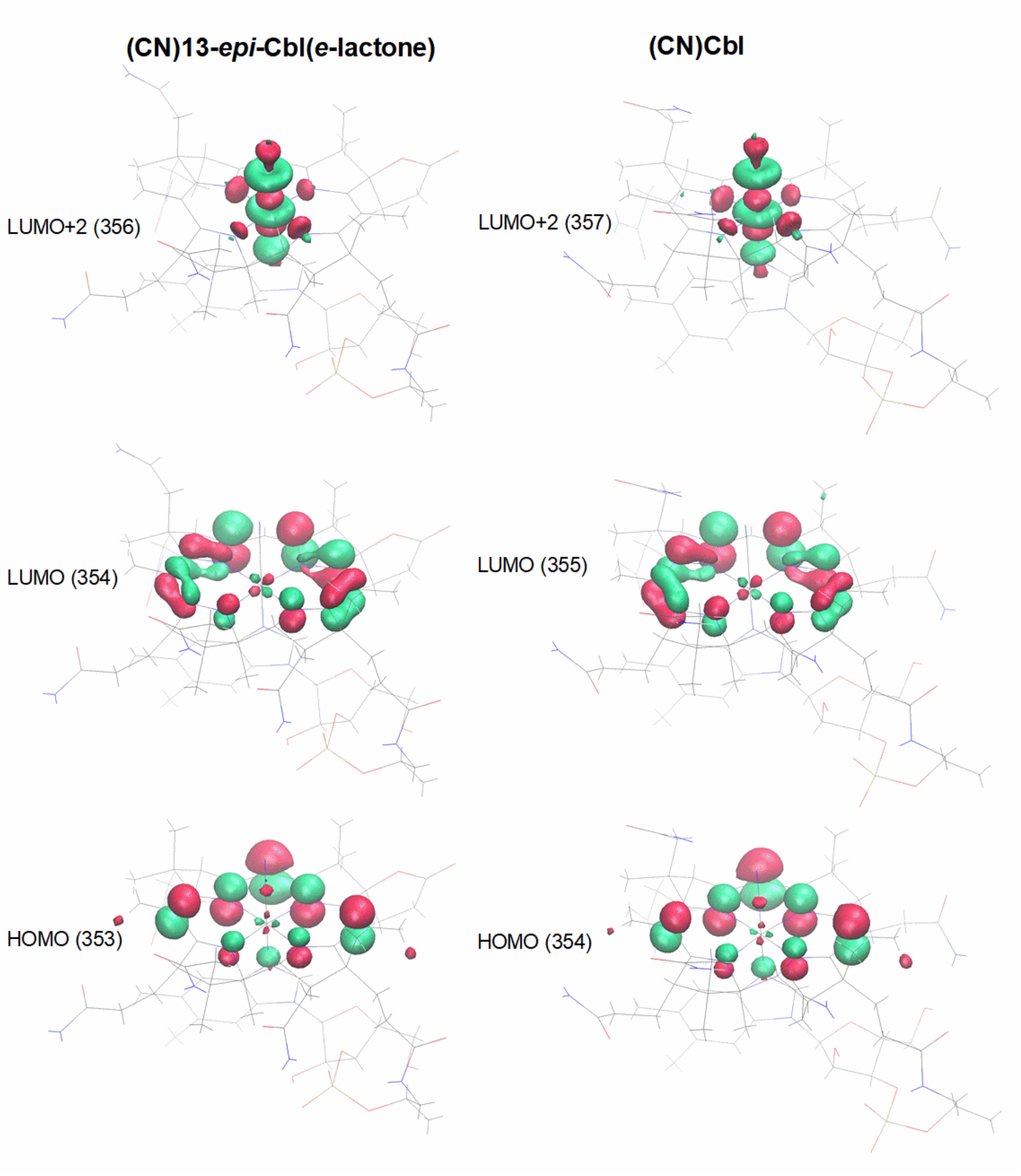

Figure S3. Molecular orbitals dominant in the first 3 electronic states of (CN)13-*epi*-Cbl(*e*-lactone) and (CN)Cbl. The VMD software was used for the plotting, with isovalue of $5 \cdot 10^{-2}$ a.u (to be continued on the next page).[6]

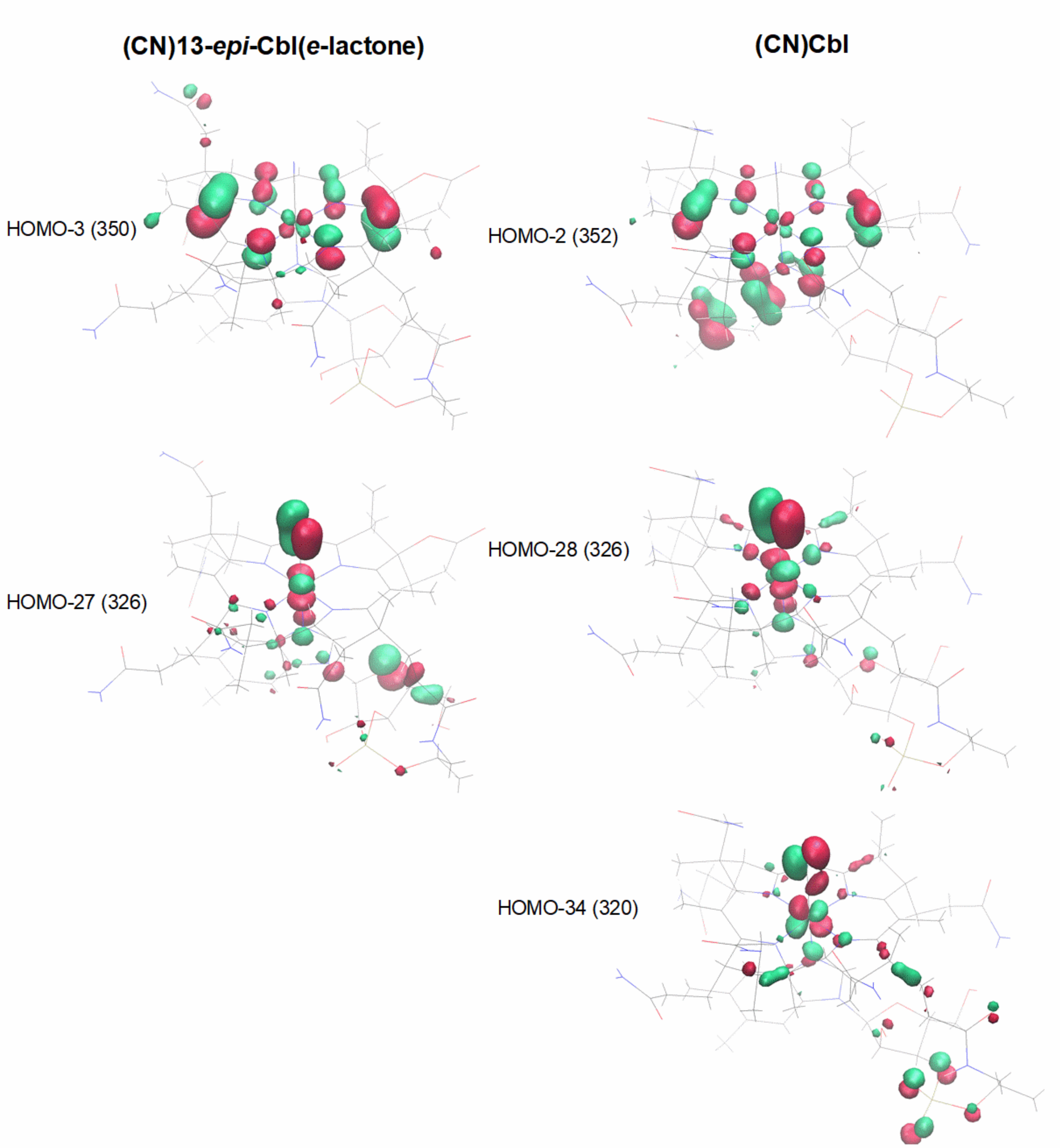

Figure S4. (continuation) Molecular orbitals dominant in the first 3 electronic states of (CN)13-*epi*-Cbl(*e*-lactone) and (CN)Cbl. The VMD software was used for the plotting, with isovalue of $5\cdot10^{-2}$ a.u.[6]

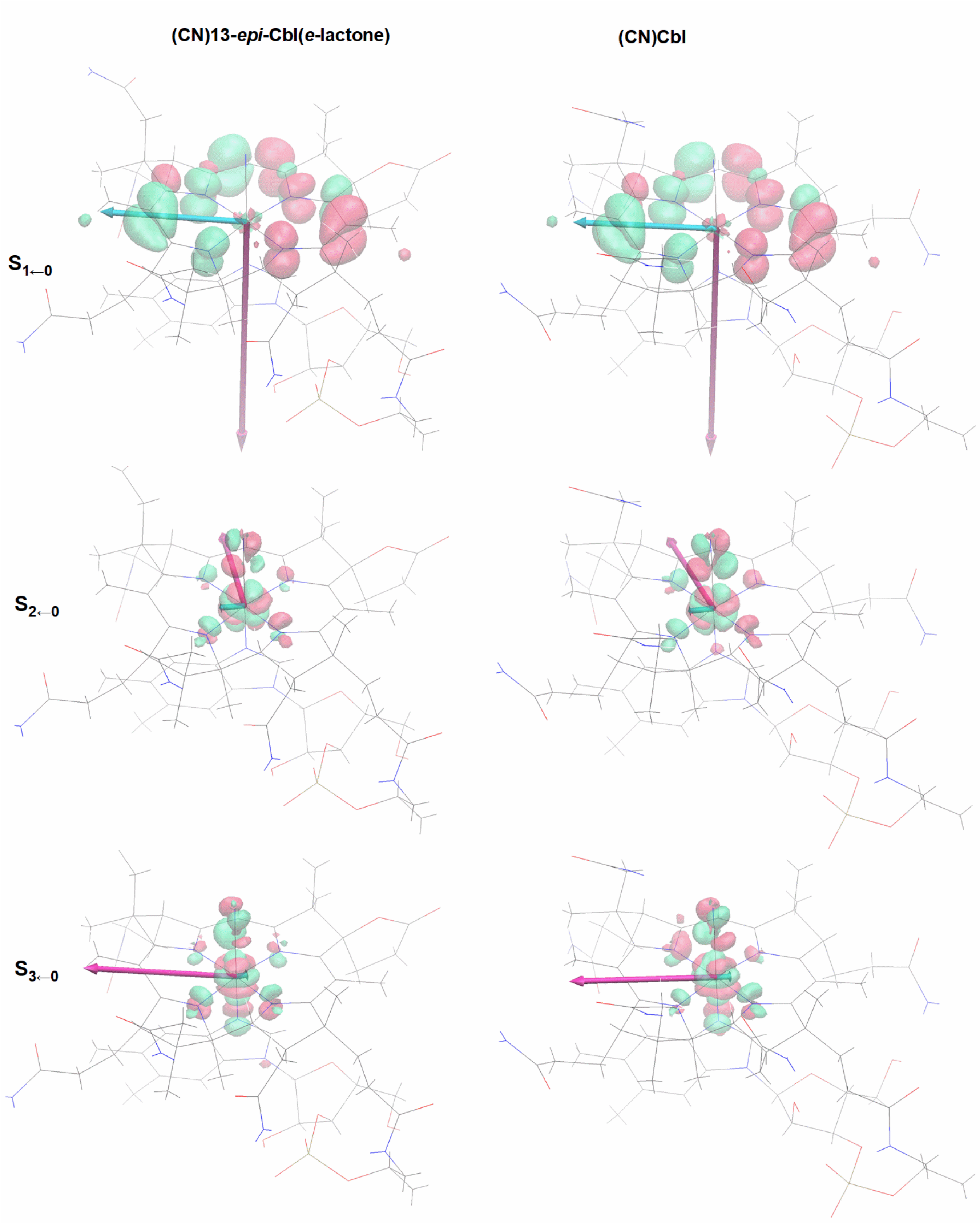

Figure S5. Transition charge densities for the first 3 electronic transitions of (CN)13-*epi*-Cbl(*e*-lactone) and (CN)Cbl. Red and green colors refer to electron and hole respectively, the isovalue is $5 \cdot 10^{-4}$ a.u. The blue and magenta arrows indicate the electric and magnetic transition dipole moments respectively. Plotted in the VMD software.[6]

ROA$_{ECD}$ calculations

ECD-ROA interference for the solute in H_2O and DMSO ((CN)Cbl or (CN)13-*epi*-Cbl(*e*-lactone)) and the solvent (DMSO) were calculated according to formula (1) and procedures described elsewhere.[9] We use experimental $\Delta\varepsilon'$ and $\Delta\varepsilon$ values of the solute, and theoretical frequencies, *DOC* factors and Raman intensities (solute or solvent), 0.2 cm pathlength L, 0.1 cm pathlength L', and experimental concentrations. The frequencies, and Raman intensities (430 nm excitation) of the solute were calculated as described above.

Experimental ROA spectra of (CN)13-*epi*-Cbl(*e*-lactone) in H_2O were reproduced as a sum of the *pre*-RROA and ROA$_{ECD}$ parts. In case of (CN)Cbl, the simulated ROA$_{ECD}$ contribution was by 1 order of magnitude smaller compared to (CN)13-*epi*-Cbl(*e*-lactone) (**Figure S7**), which is consistent with the experiment (**Figure 2**).

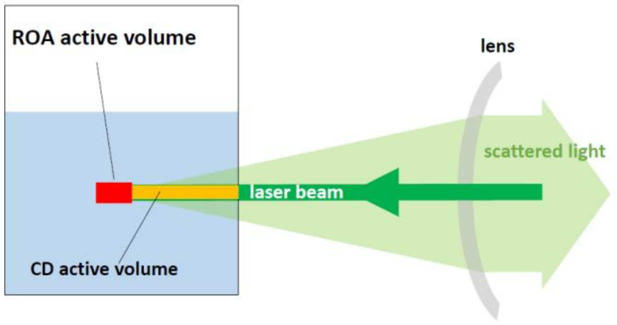

Figure S6. Laser focusing scheme in the ROA experiment.

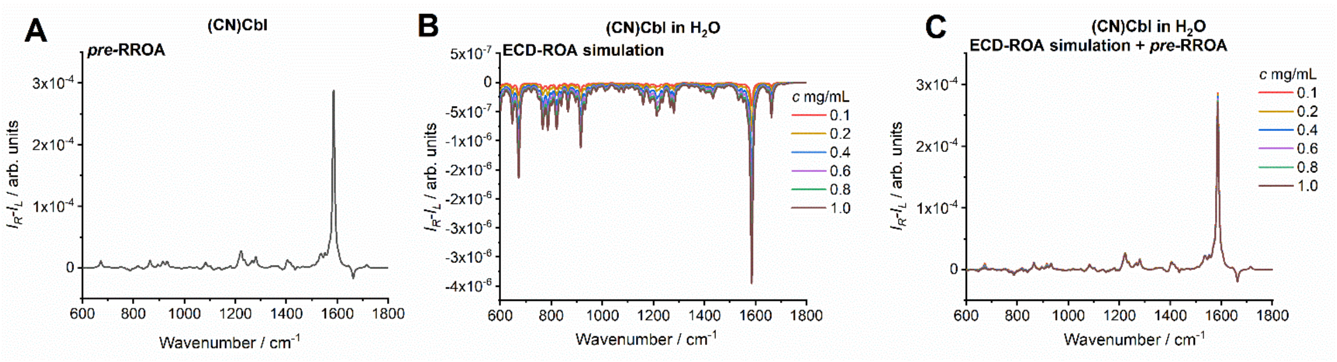

Figure S7. Theoretical ROA spectra of (CN)Cbl: *pre*-resonance ROA calculated at 430 nm excitation wavelength (**A**); ROA$_{ECD}$ simulation calculated for a variety of concentrations in use of formula (1) (**B**); sum of ROA$_{ECD}$ and pre-RROA spectra(**C**).

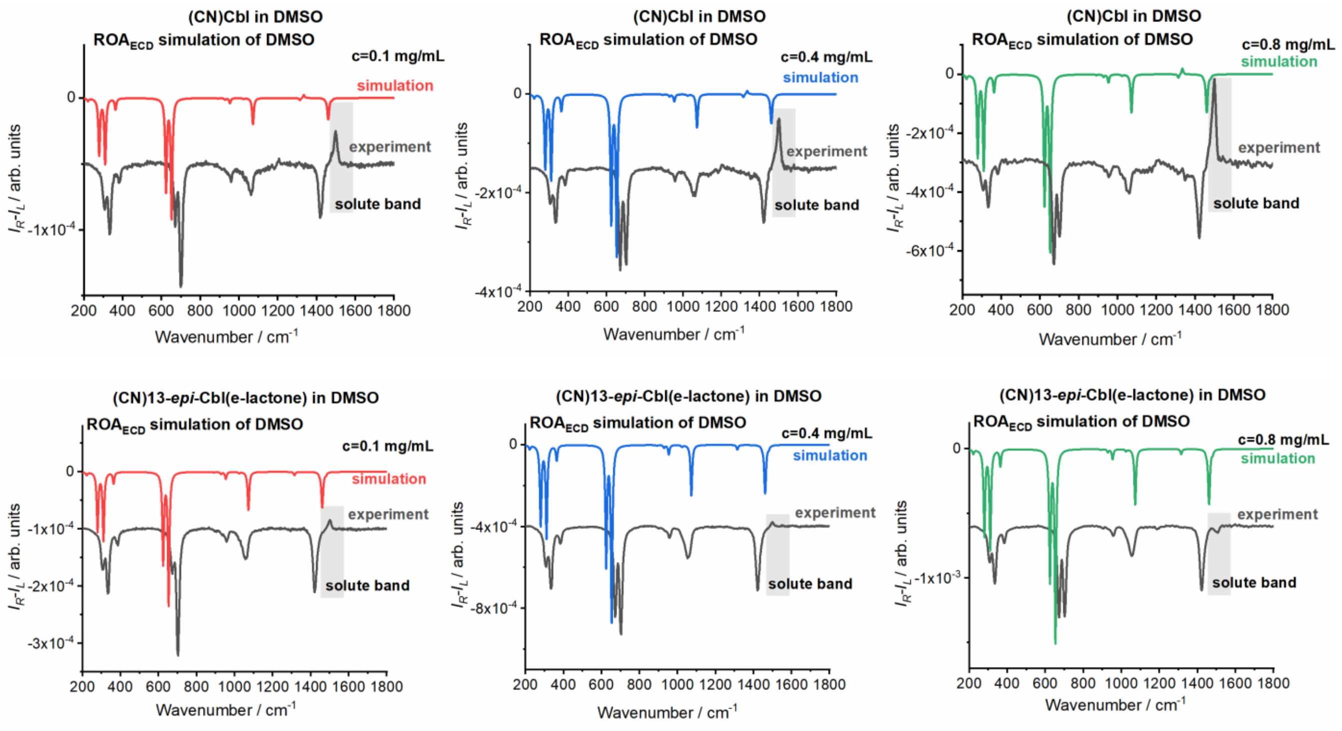

Figure S8. Experimental ROA spectra of (CN)Cbl (top) and (CN)13-*epi*-Cbl(*e*-lactone) (bottom), compared with calculated ROA$_{ECD}$ signal of DMSO for three different concentrations.

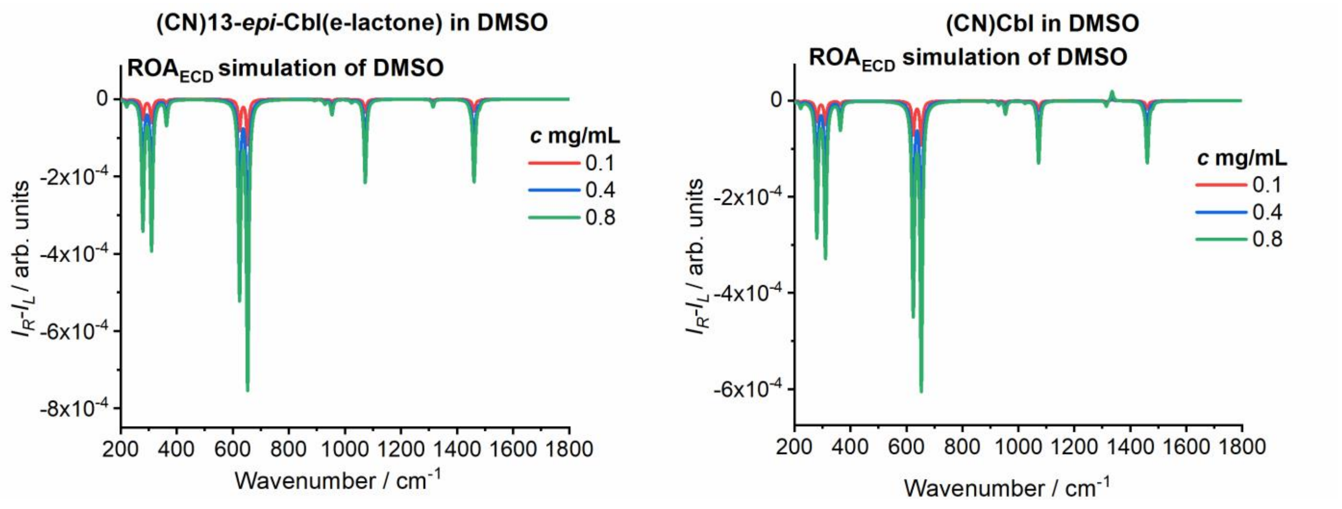

Figure S9. The ROA$_{ECD}$ signal of DMSO spectra for (CN)13-*epi*-Cbl(*e*-lactone) and (CN)Cbl in DMSO systems, calculated for a variety of concentrations.

Experimental Procedures

Materials

Cyanocobalamin ((CN)Cbl, vitamin B_{12}) and dimethyl sulfoxide (DMSO) were purchased from Sigma-Aldrich and were used as received. Chemical derivatives of vitamin B_{12}, such as (CN)13-*epi*-Cbl, (CN)13-*epi*-Cbl(*e*-lactone), (CN)13-*epi*-Cbl(*e*-CO_2Me)(13-OH), (CN)Cbl(10-NO_2), (CN)Cbl(10-NH_2) and (CN)Cbl(*c*-lactone) were synthesized according to reported procedures.[10–13] The modified procedure for the synthesis of (CN)13-*epi*-Cbl was described in the section **Organic Synthesis** (page 27).

Methods

Electronic Absorption (UV-Vis) and Electronic Circular Dichroism (ECD) measurements

UV-Vis and ECD spectra of vitamin B_{12} and its derivatives were recorded in the 230-650 nm spectral range at room temperature in distilled water or DMSO. The solutions in various concentrations were measured in the 10, 2 and 1 mm quartz cells. All spectra were recorded in a single scan on Jasco J−1500 spectropolarimeter with 100 nm min^{-1} scanning speed, step size of 0.2 nm, 1 nm bandwidth, and a response time of 1 s. They were background-corrected using solvents recorded under the same conditions. UV-Vis/ECD spectra of studied samples were also measured after Raman/ROA experiments to control sample stability. Because of the limited amount of solution (~140 μL), in this case they were registered in quartz optical cell with a path length of 1 mm and accumulation of 3-5 scans (**Figure 1**, **S11** and **S13**).

Raman and Raman Optical Activity (ROA) measurements

Raman and ROA spectra of vitamin B_{12} and its analogs dissolved in distilled water as well as DMSO were obtained on a ChiralRAMAN-2X™ spectrometer (BioTools Inc.) at 7 cm^{-1} resolution within 250- 2500 cm^{-1} employing the excitation wavelength of 532 nm. Before starting the measurements, all solutions were passed through the Millex® (Merck MilliporeTM) syringe PTFE filters (pore size 0.2 μm) to eliminate solid impurities.

Various concentrations (from 0.1 to 0.8 mg/mL) were measured in ROA quartz optical cells with anti-reflective coating. A laser power of 200 mW and integration time of 4 s were used for samples in water. For compounds measured in DMSO at 0.1 mg/mL concentration, laser power of 100 mW and the integration time of 1 s were employed. Specific conditions for each sample are given in **Table S5**.

We emphasis that Raman/ROA spectra were registered with long (all samples listed in **Table S5**) and short path lengths. The measurements using the short path length included aqueous solutions of (CN)13-*epi*-Cbl(*e*-lactone) at 0.1 and 0.8 mg/mL concentrations (**Figure 5** and **S22**). To prevent degradation, for most of the samples, many Raman/ROA measurements of freshly prepared solutions were averaged (**Table S5**). Minor baseline corrections of both Raman and ROA was also applied (**Figure 3**, **6** and **S24, S25**). Raw spectra are provided below (**Figures S13-S22**). At least three independent experiments for each sample were conducted, but the obtained ROA spectra were quite reproducible (**Figure S24**).

Table S5. Set of measurement parameters of studied compounds dissolved in water or DMSO.

Compound	Concentration [mg/mL]	Total data collection time [h]	Total measurement number of freshly prepared solutions taken for average
(CN)Cbl	0.1	24	1
	0.2	24	2
	0.4	24	1
	0.6	26	2
	0.8	24	1
	1	24	1
(CN)13-*epi*-Cbl	0.1	46	4
	0.5	55	5
	0.8	60	6
(CN)13-*epi*-Cbl(*e*-lactone)	0.1	48	4
	0.2	72	6
	0.4	74	3
	0.6	48	4
	0.8	40	2
	1	47	2
(CN)13-*epi*-Cbl(*e*-CO_2Me)(13-OH)	0.1	54	5
	0.4	64	6
	0.8	38	3
(CN)Cbl(10-NO_2)	0.1	25	25
	0.3	18	18
	0.8	20	20
(CN)Cbl(10-NH_2)	0.1	26	8
	0.8	22	7
	1.2	20	7
(CN)Cbl(*c*-lactone)	0.1	31	10
	0.8	24	8
(CN)Cbl **in DMSO**	0.1	24	2
	0.4	26	2
	0.8	27	2
(CN)13-*epi*-Cbl(*e*-lactone) **in DMSO**	0.1	24	2
	0.4	31	2
	0.8	27	2

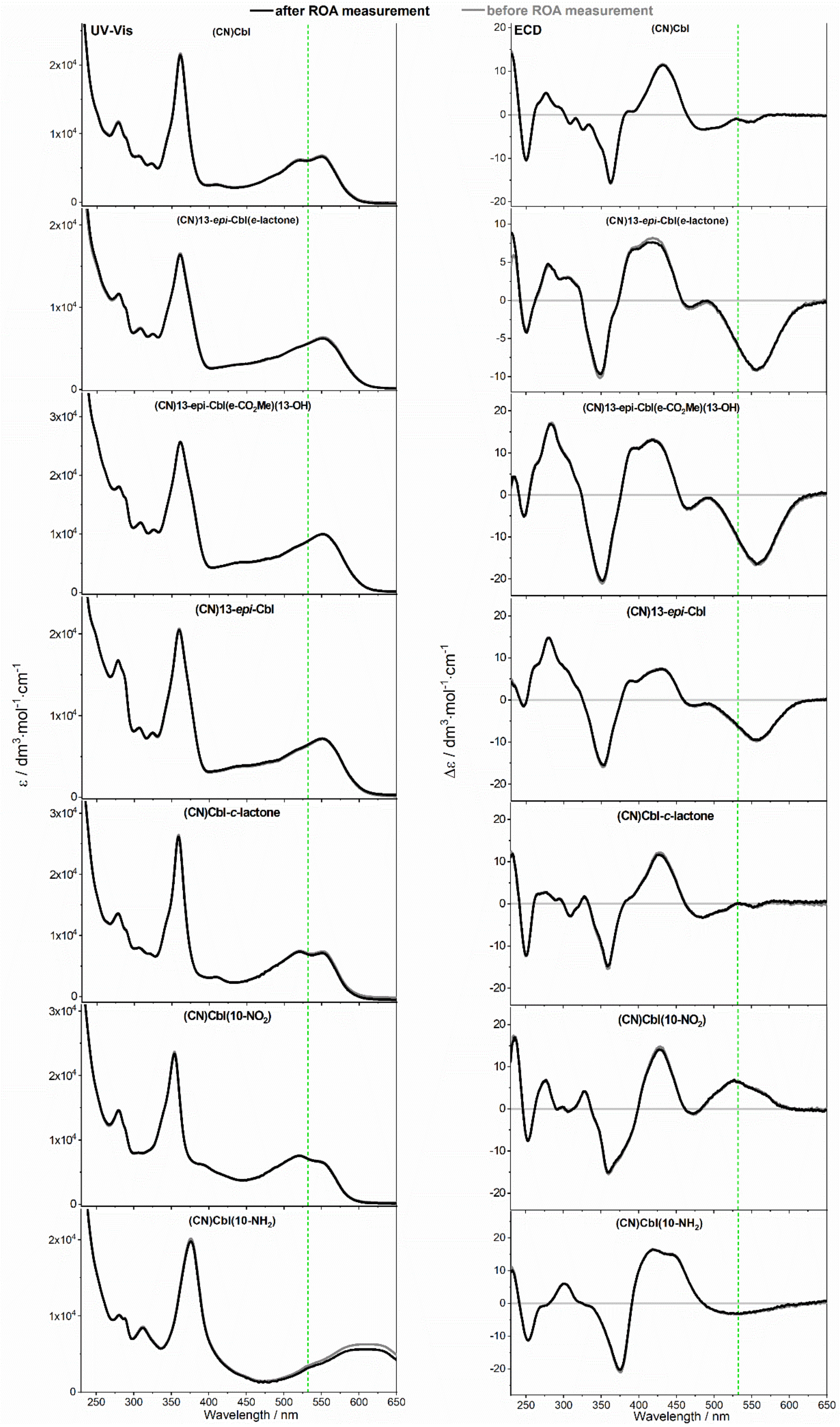

Figure S10. UV-Vis and ECD spectra of vitamin B_{12} and its analogues dissolved **in water** at 0.8 mg/mL concentration recorded before (grey line) and after (black line) single ROA experiments involving laser irradiation.

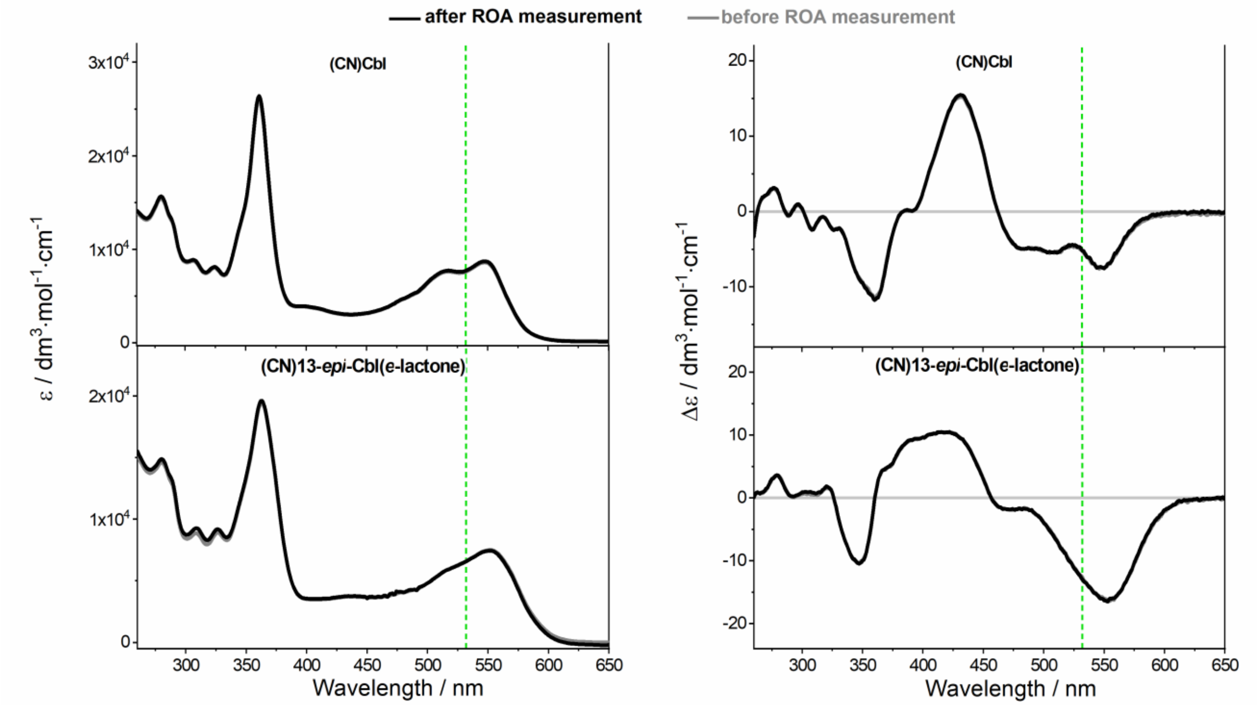

Figure S11. UV-Vis and ECD spectra of vitamin B_{12} and (CN)13-*epi*-Cbl(*e*-lactone) dissolved **in DMSO** at 0.8 mg/mL concentration recorded before (grey line) and after (black line) single ROA experiments.

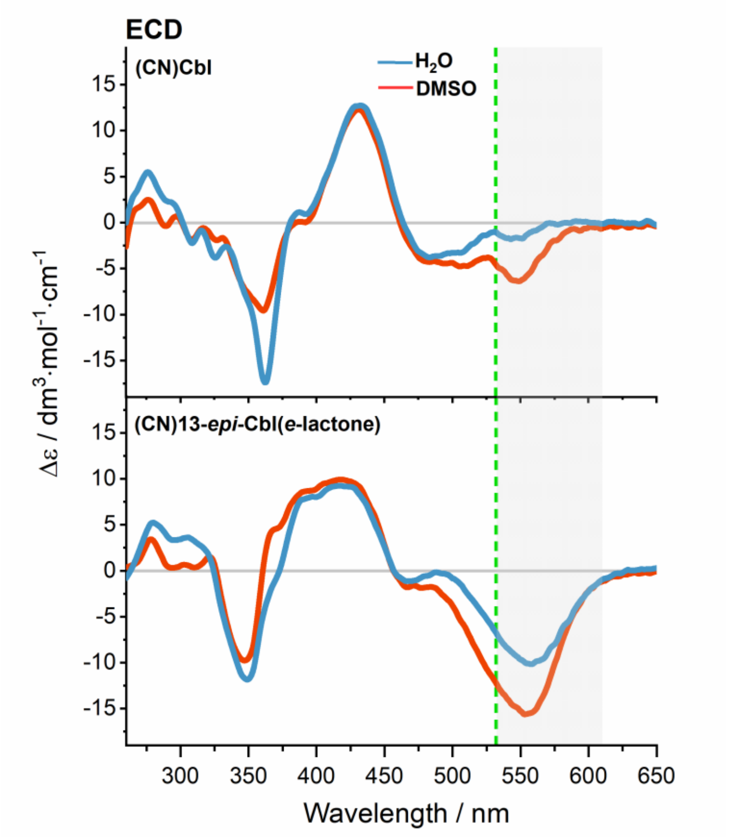

Figure S12. ECD spectra of (CN)Cbl and (CN)13-*epi*-Cbl(*e*-lactone) measured for water (blue line) and DMSO (red line) at 0.8 mg/mL concentration. The grey belts indicate the spectral range from 532 to 610 nm, that corresponds to the range 0-2400 cm^{-1} in Raman and ROA spectra.

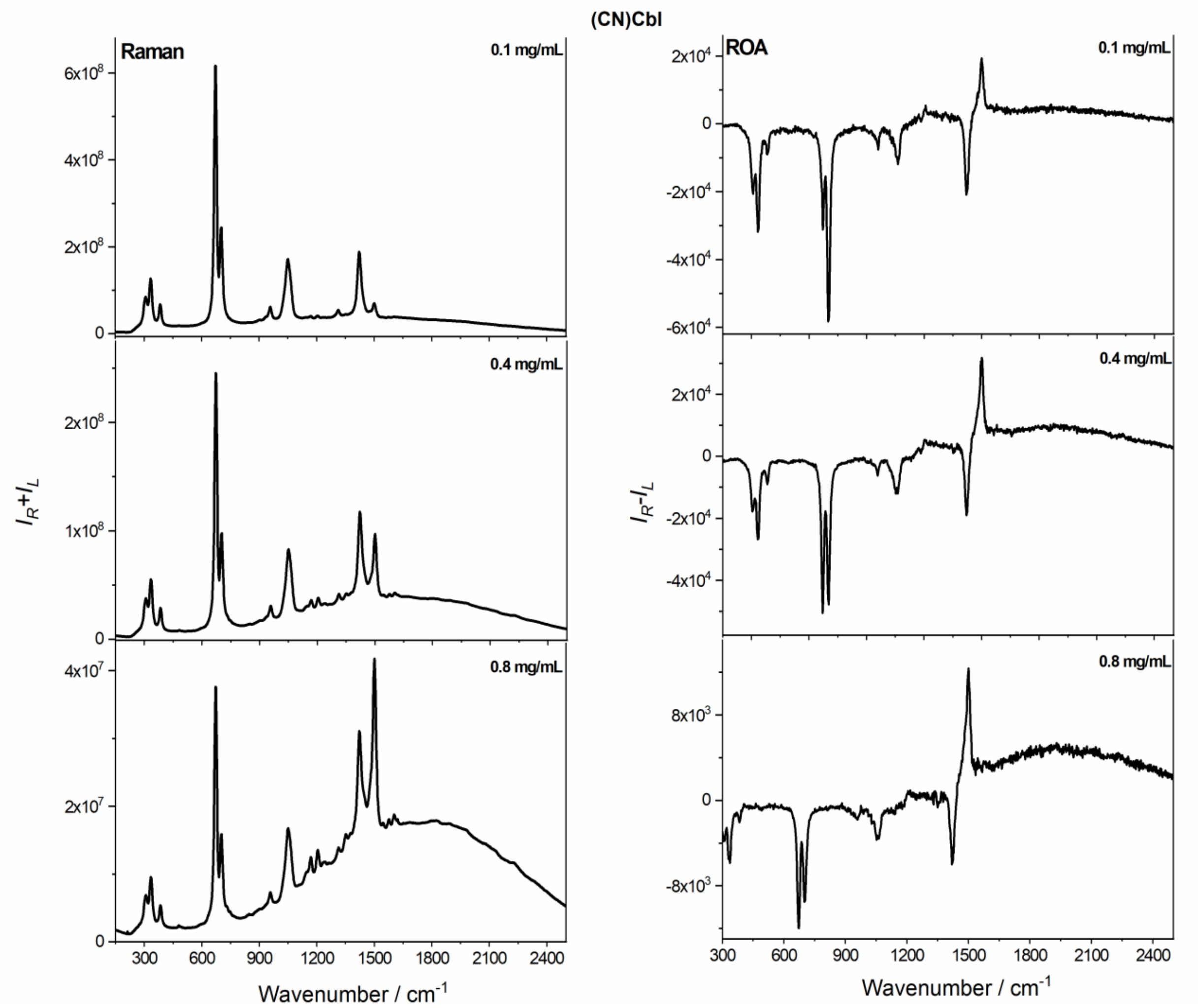

Figure S13. Raw Raman and ROA spectra of vitamin B_{12} dissolved **in DMSO** at different concentrations.

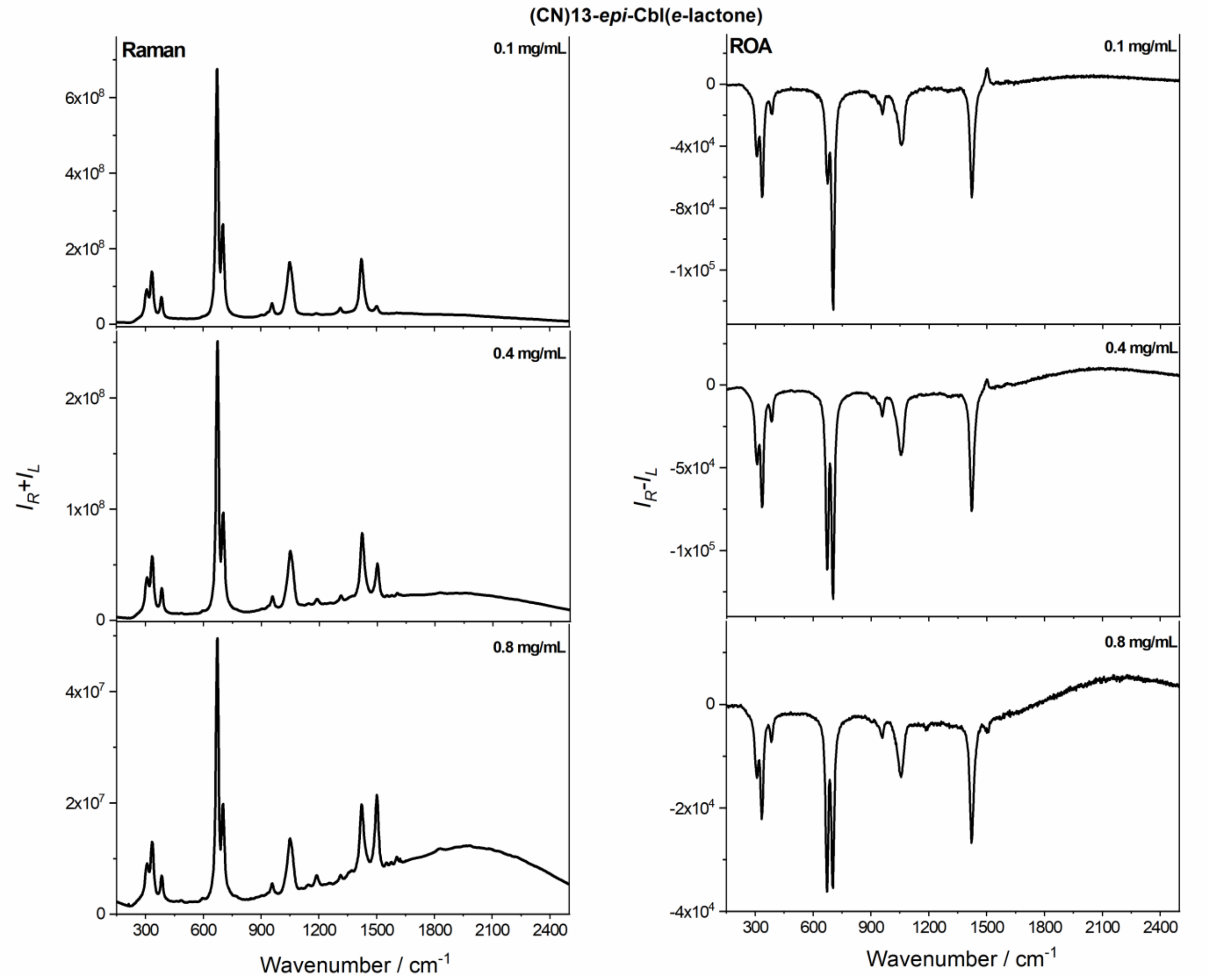

Figure S14. Raw Raman and ROA spectra of (CN)13-*epi*-Cbl(*e*-lactone) dissolved **in DMSO** at different concentrations.

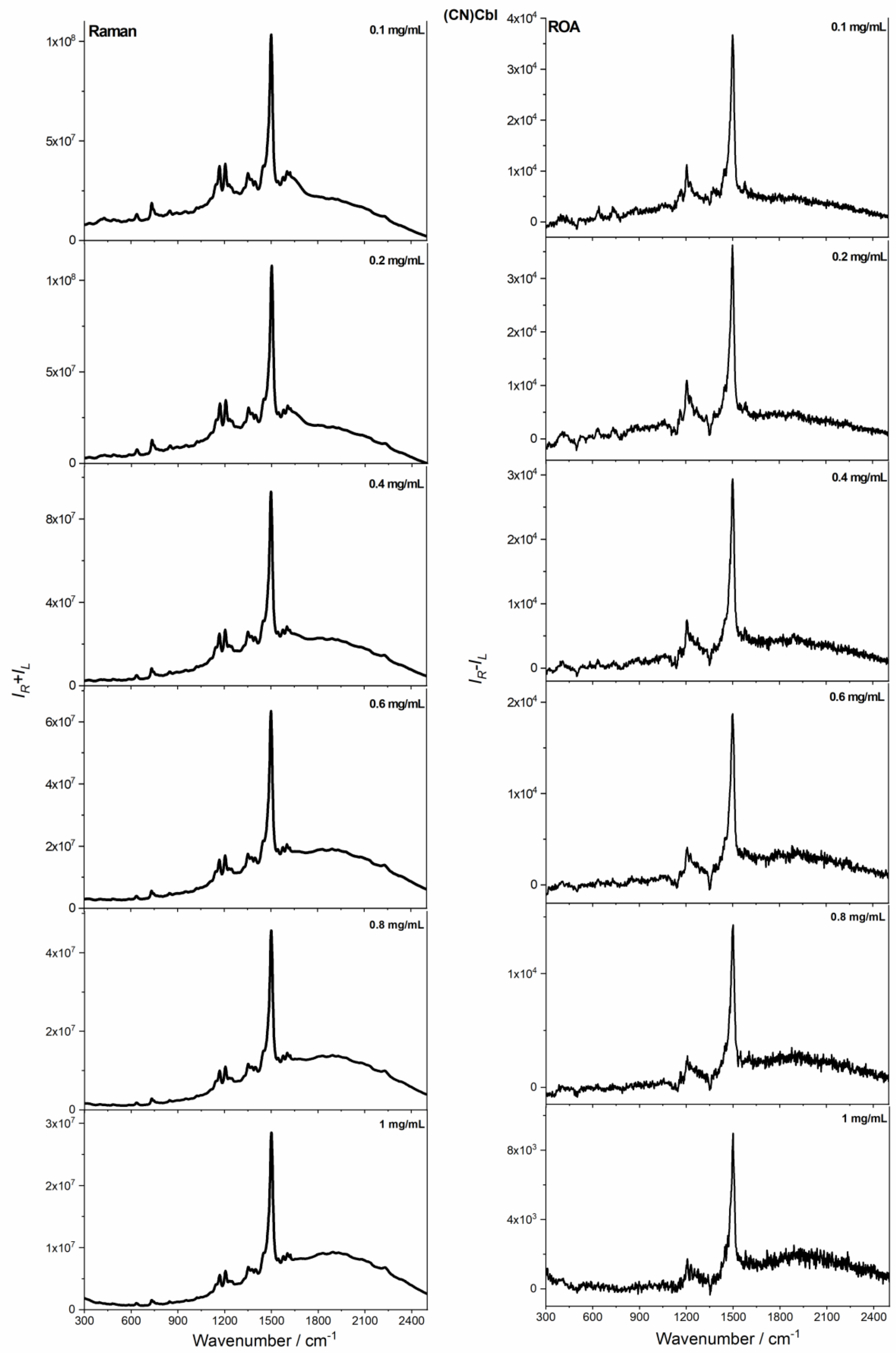

Figure S15. Raw Raman and ROA spectra of vitamin B_{12} dissolved **in water** at different concentrations.

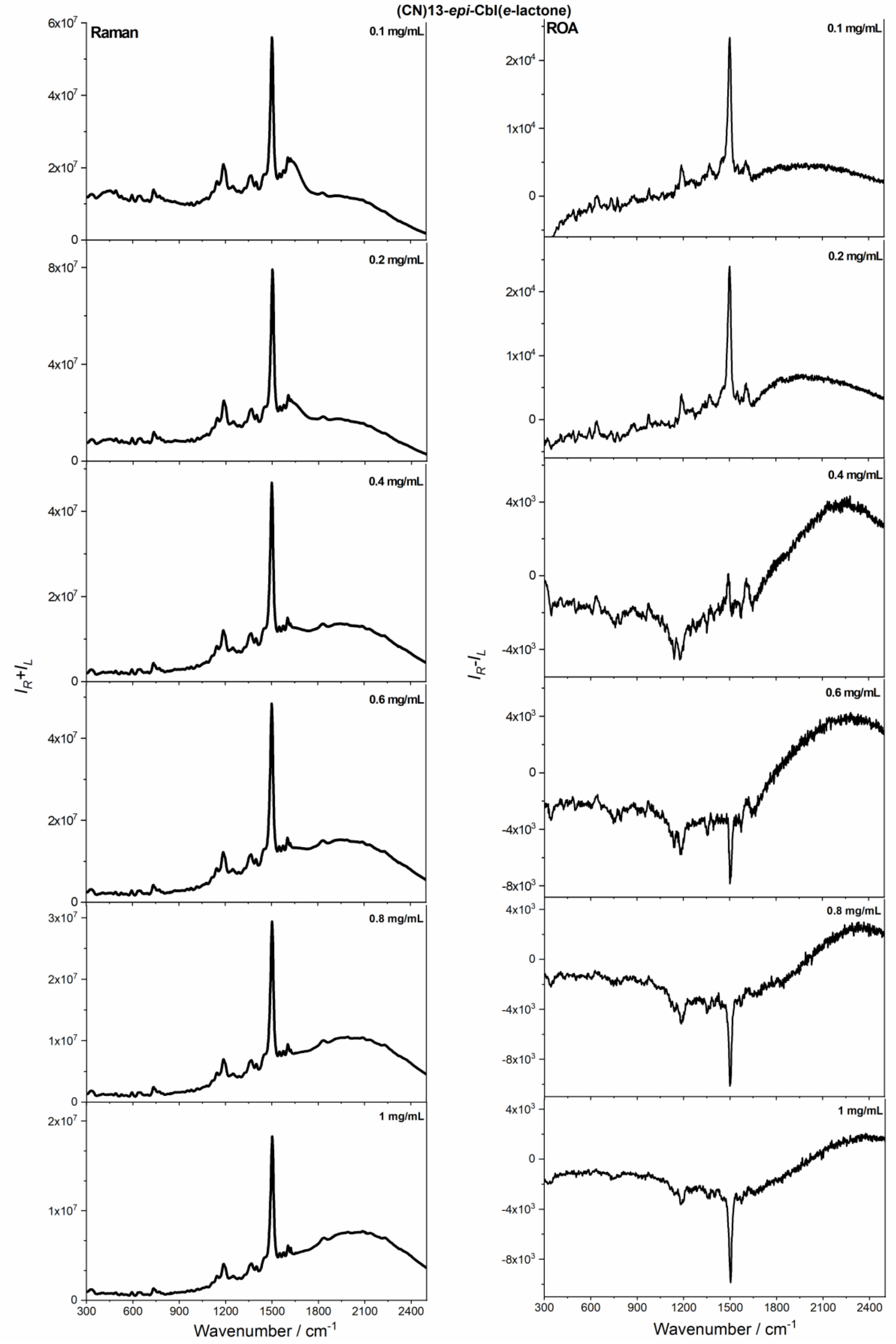

Figure S16. Raw Raman and ROA spectra of (CN)13-*epi*-Cbl(*e*-lactone) dissolved **in water** at different concentrations.

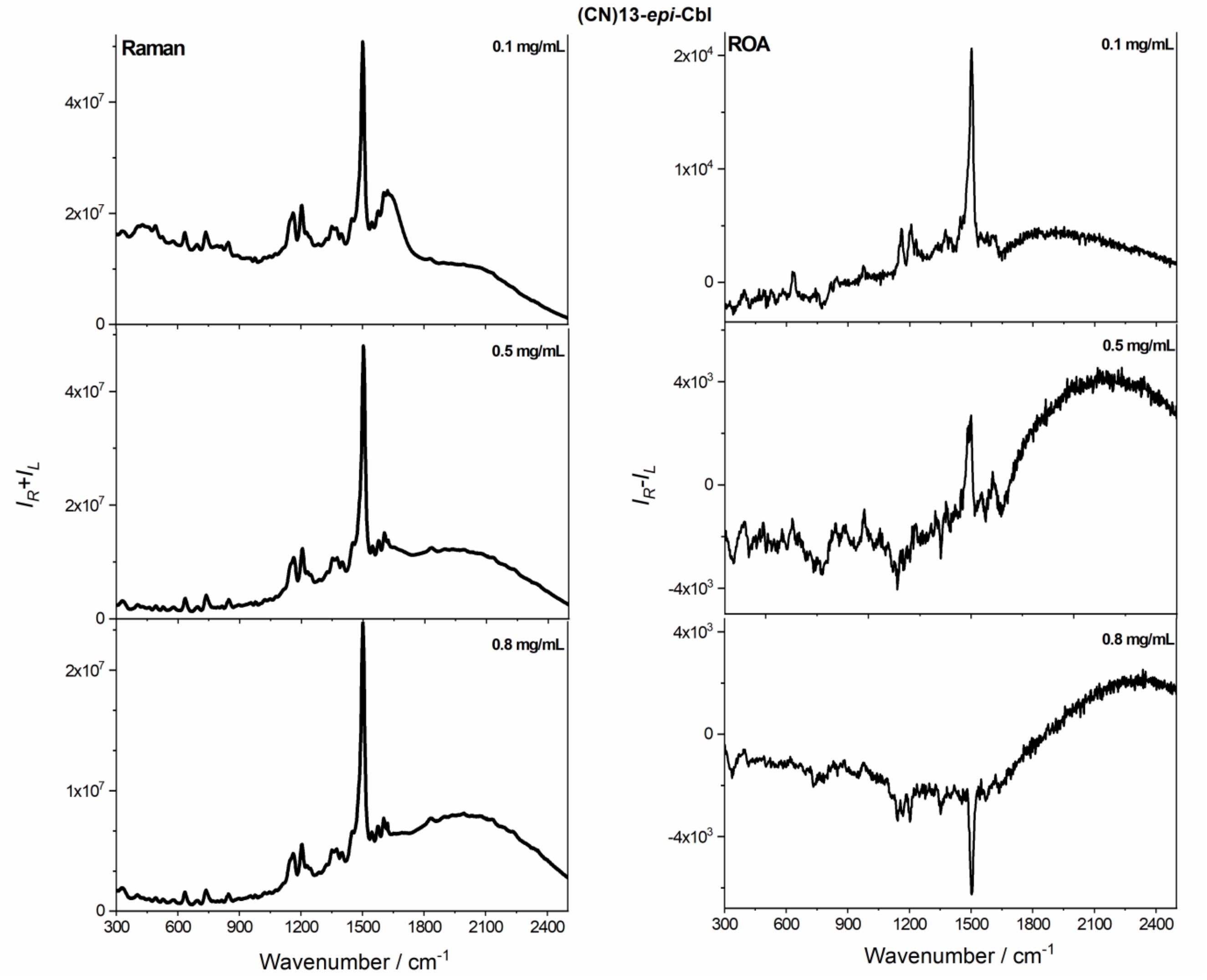

Figure S17. Raw Raman and ROA spectra of (CN)13-*epi*-Cbl dissolved **in water** at different concentrations.

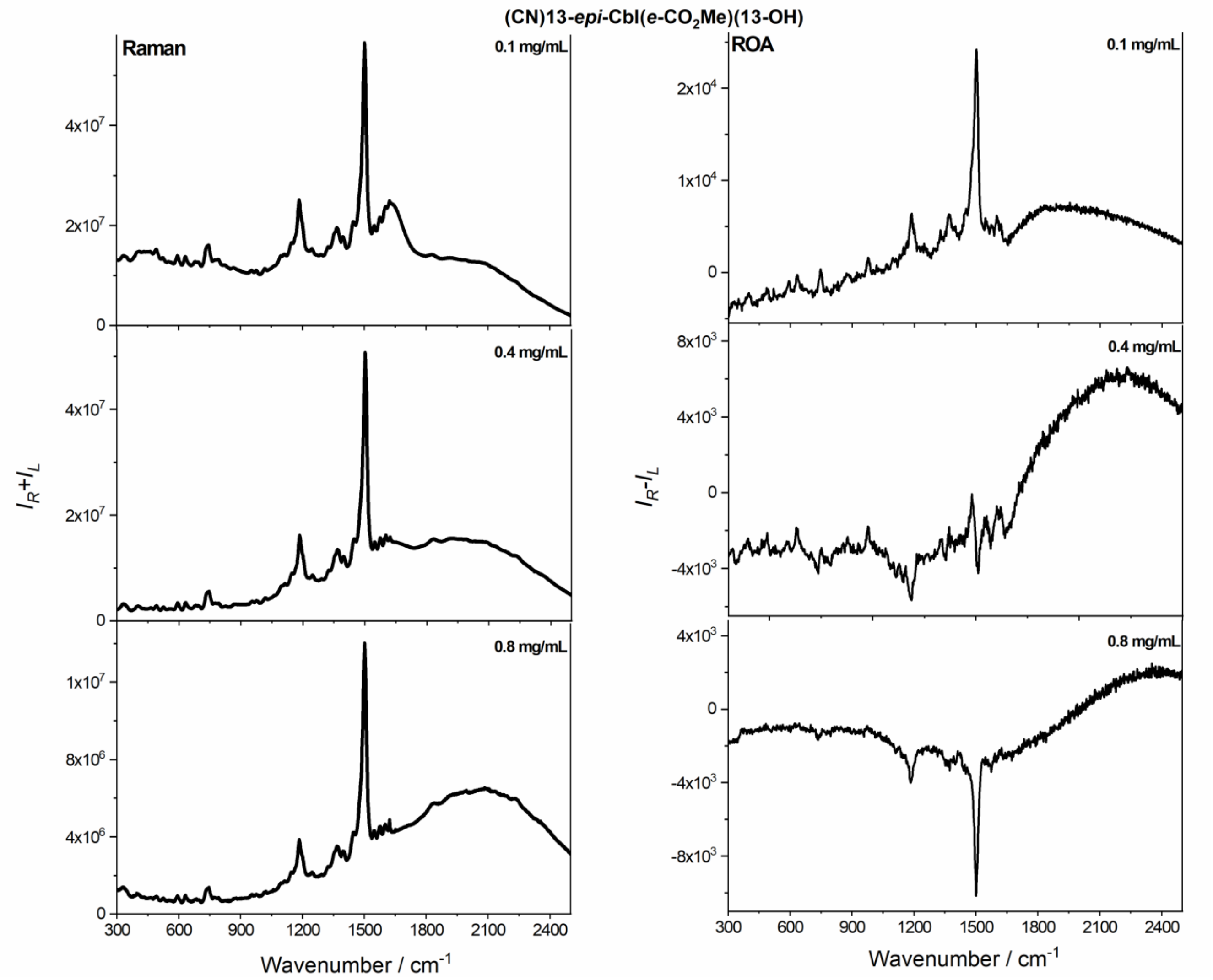

Figure S18. Raw Raman and ROA spectra of (CN)13-*epi*-Cbl(*e*-CO_2Me)(13-OH) dissolved **in water** at different concentrations.

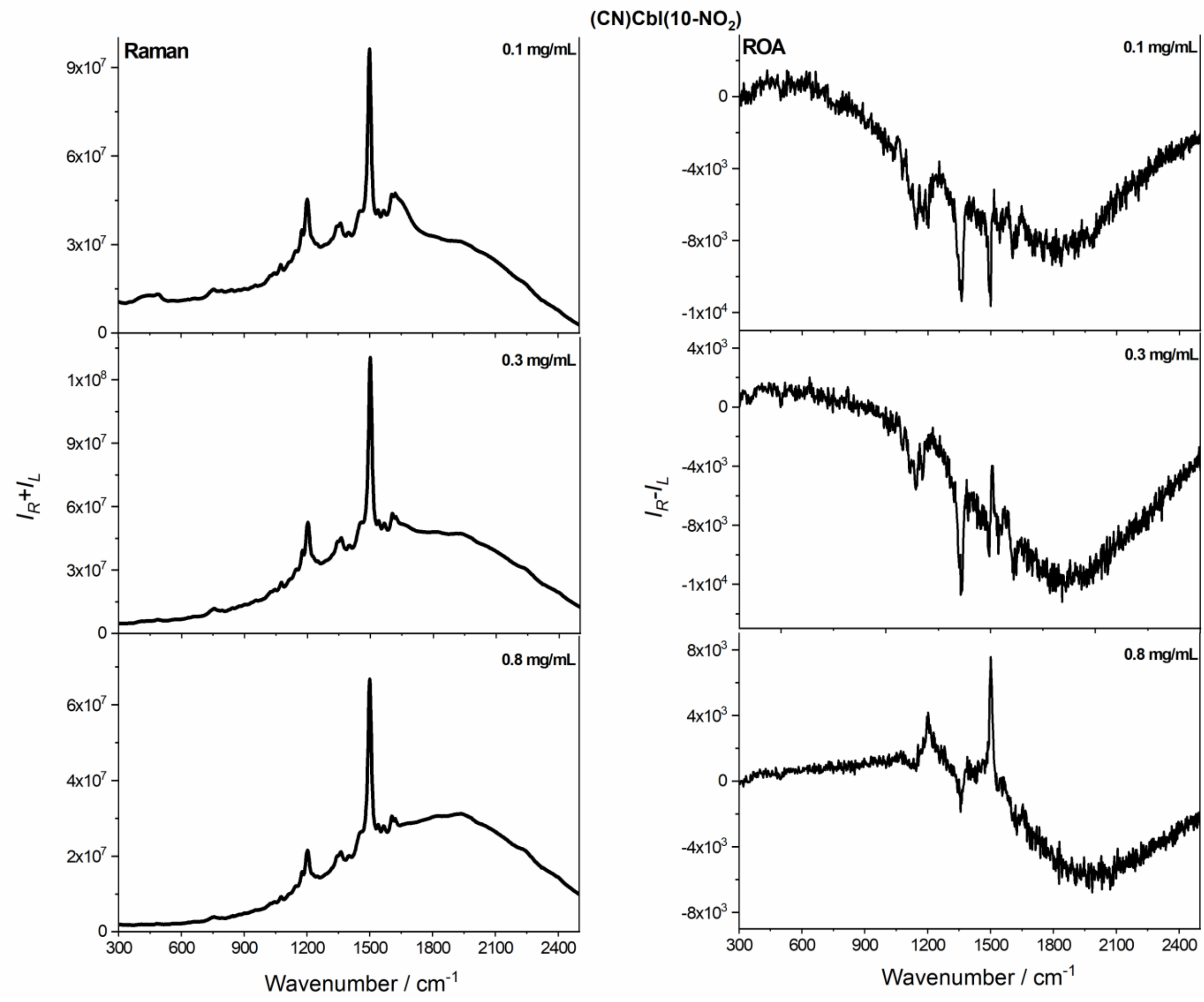

Figure S19. Raw Raman and ROA spectra of (CN)Cbl(10-NO_2) dissolved **in water** at different concentrations.

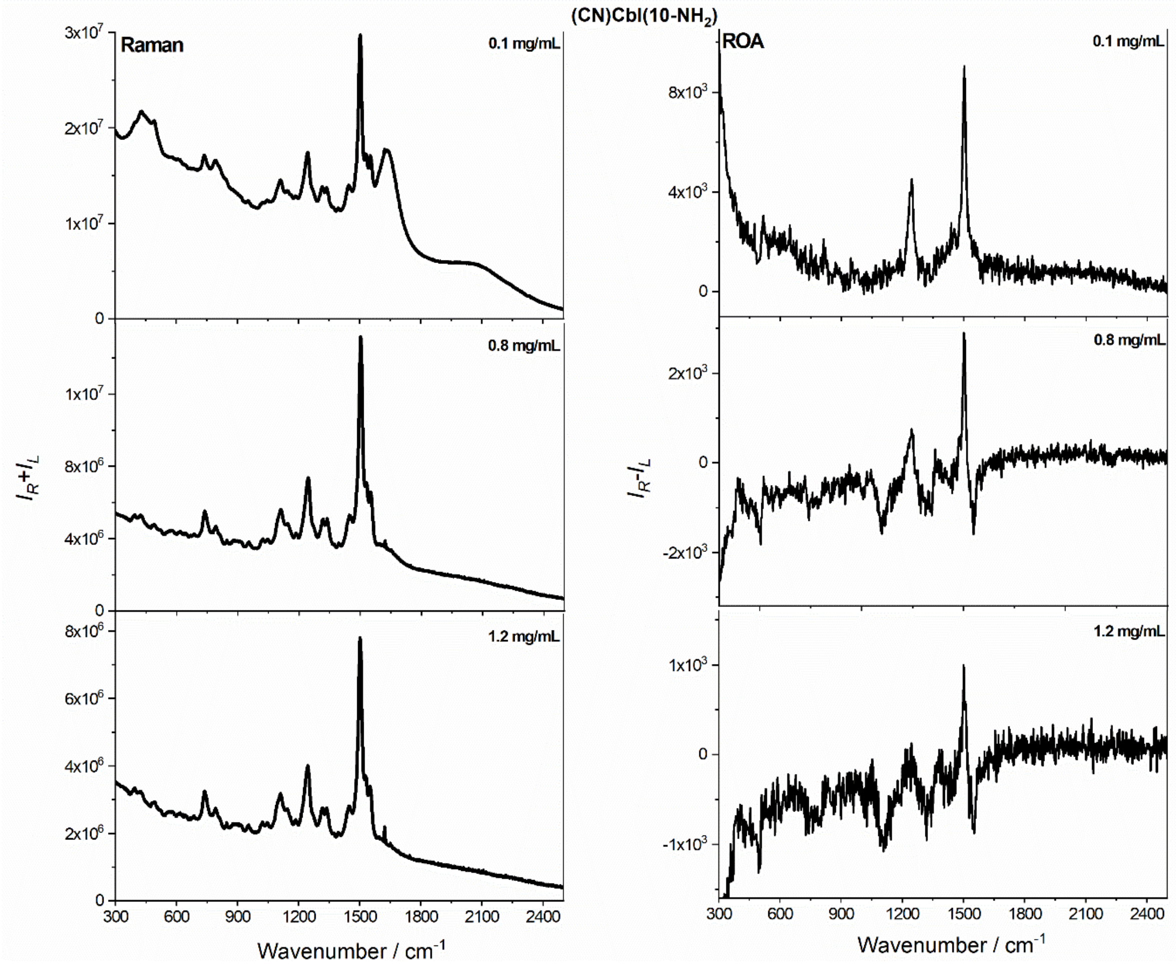

Figure S20. Raw Raman and ROA spectra of (CN)Cbl(10-NH_2) dissolved **in water** at different concentrations.

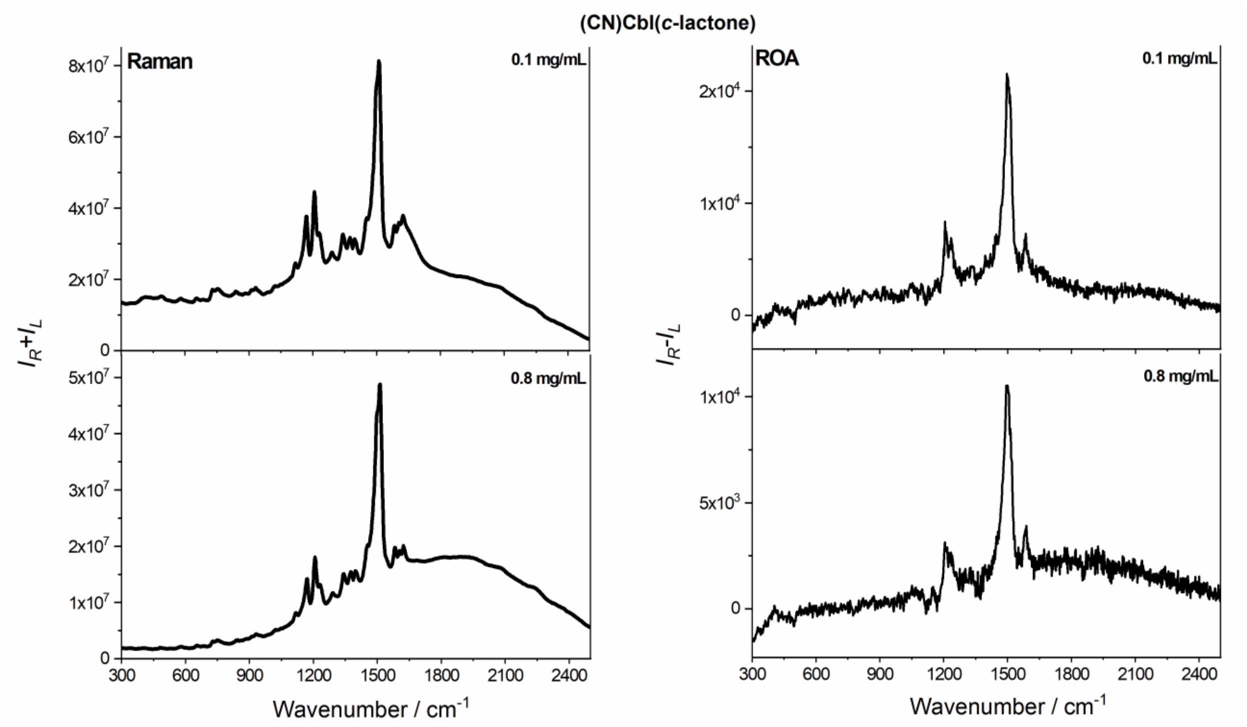

Figure S21. Raw Raman and ROA spectra of (CN)Cbl(*c*-lactone) dissolved **in water** at different concentrations.

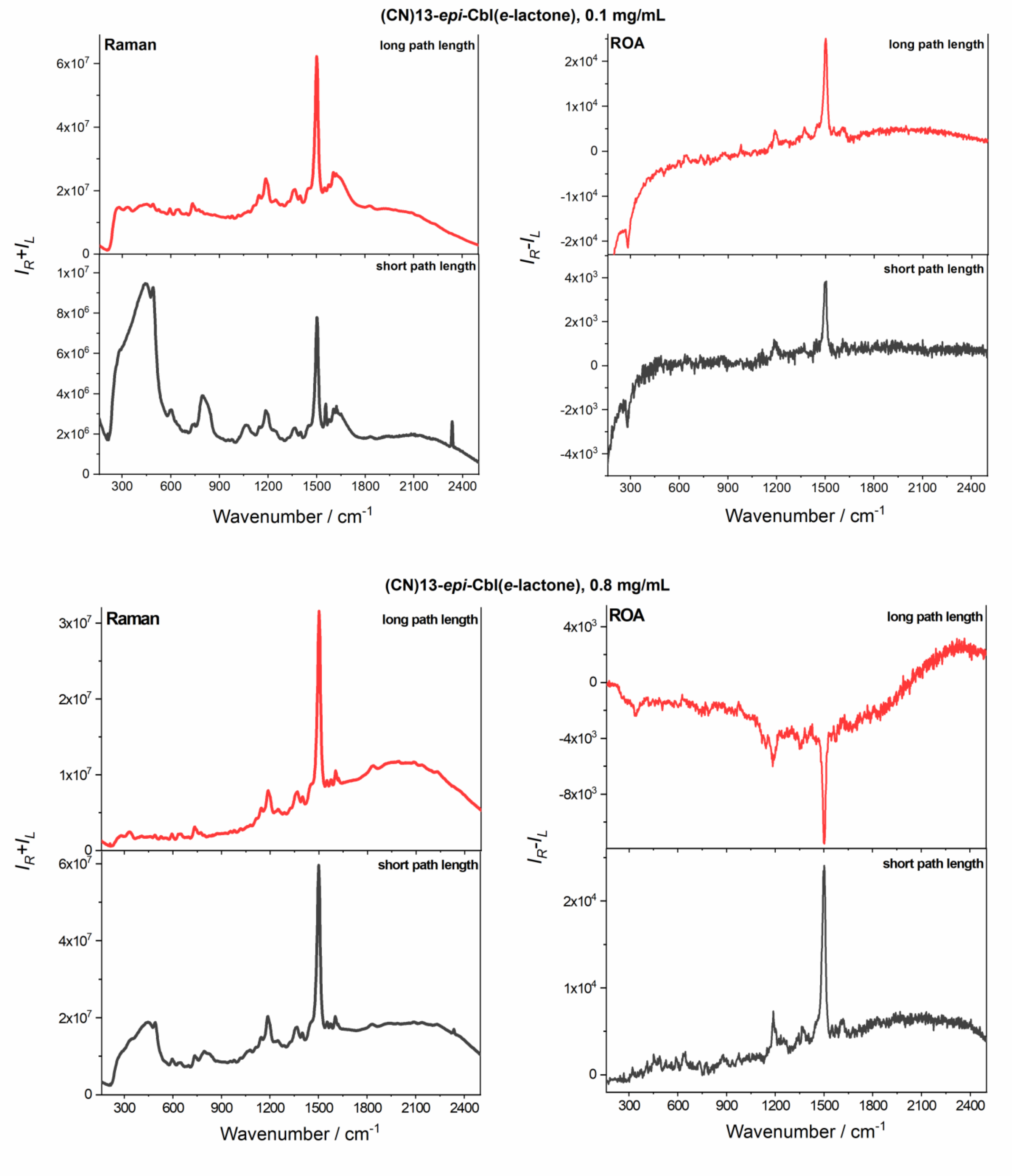

Figure S22. Raw Raman and ROA spectra of (CN)13-*epi*-Cbl(*e*-lactone) dissolved **in water** at 0.1 and 0.8 mg/mL concentrations. The spectra were registered with a long and a short path length.

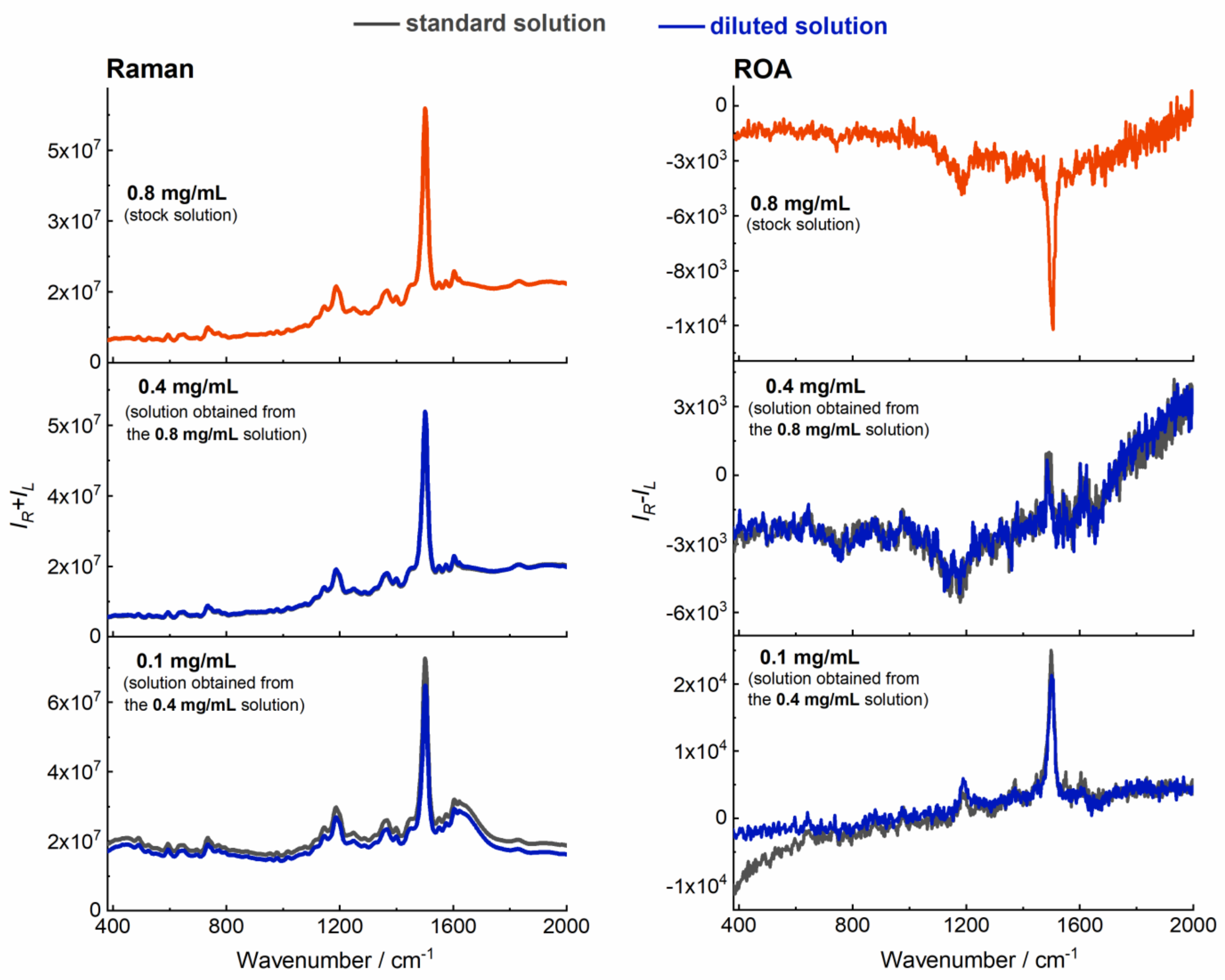

Figure S23. Raw Raman and ROA spectra of (CN)13-*epi*-Cbl(*e*-lactone) in water at different concentrations (from 0.8 to 0.1 mg/mL). The blue spectra were obtained with the stock solution with concentration of 0.8 mg/mL that was measured, diluted to 0.4 mg/mL, measured once more, etc. The black spectra were measured with fresh samples.

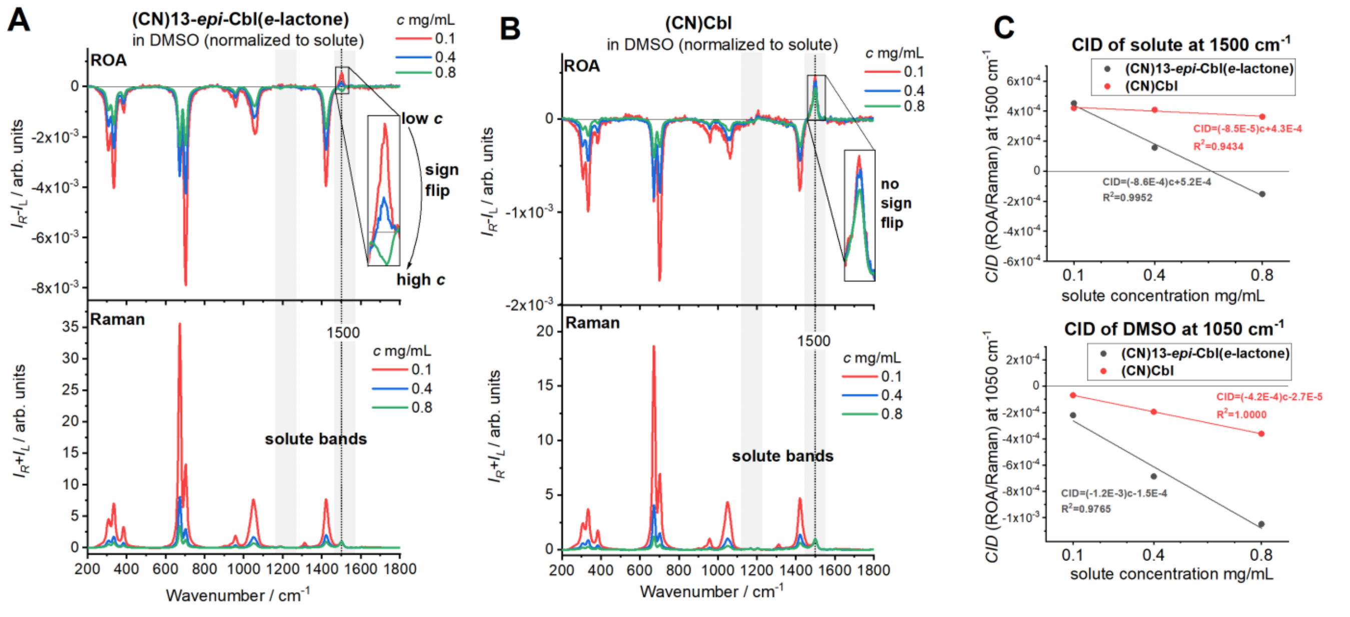

Figure S24. ROA and Raman spectra of (CN)Cbl (A) and (CN)13-*epi*-Cbl(*e*-lactone) (B) measured for DMSO, normalized to the solute's maximum Raman band in order to preserve CID ratios of each measurement; comparison of CID values vs concentration for (CN)Cbl and (CN)13-*epi*-Cbl(*e*-lactone) (C).

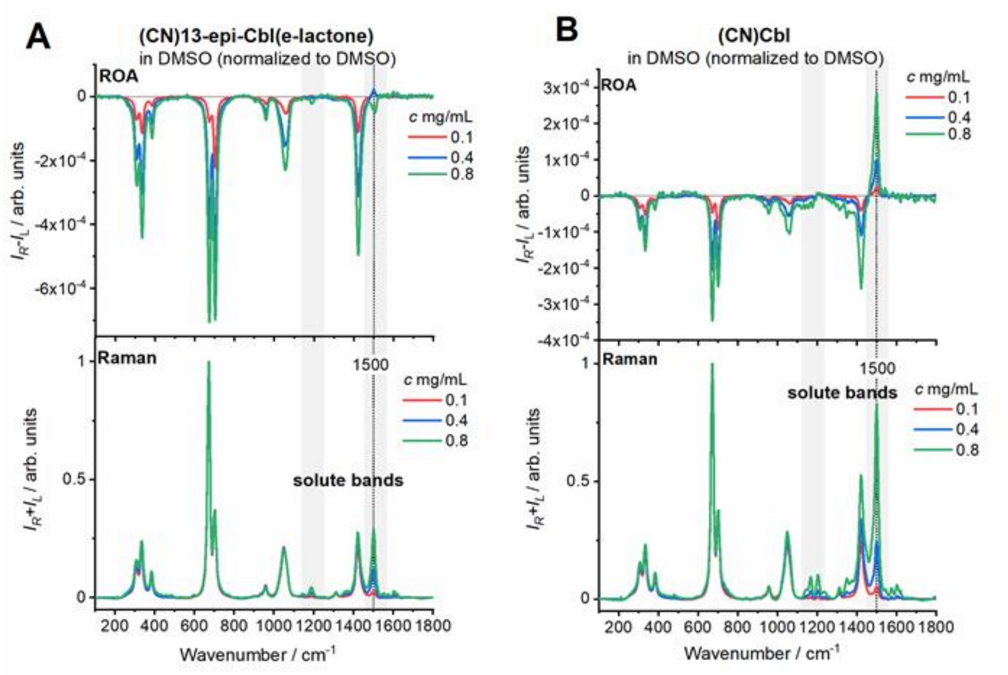

Figure S25. ROA and Raman spectra of (CN)13-*epi*-Cbl(*e*-lactone) (A) and (CN)Cbl (B) measured in DMSO, normalized to the strongest Raman band while preserving CID ratios.

Organic synthesis

The procedure for the synthesis of (CN)13-*epi*-Cbl was repeated with the following changes:
(CN)Cbl (0.221 mmol, 300 mg) was suspended in trifluoroacetic acid (5.8 mL) and the mixture was stirred at room temperature for 2 h. Subsequently the acid was removed under reduced pressure and the residue was dried in vacuo for 20 min. The crude product was suspended in diethyl ether, mixed thoroughly, filtered and washed with another portion of diethyl ether. The red solid was redissolved in water and purified via reversed-phase column chromatography using LiChroprep RP-18 (40–63 mm) silica with redistilled water and HPLC-grade CH_3CN as eluents (gradually form 5% to 15% CH_3CN/H_2O, v/v). The first intense red band was collected and concentrated in vacuo. The pure (CN)13-*epi*-Cbl was isolated as a red solid (36 mg, 12% yield).

^{1}H NMR (600 MHz, CD_3OD) δ 7.27 (s, 1H), 7.19 (s, 1H), 6.56 (s, 1H), 6.27 (d, *J* = 3.2 Hz, 1H), 5.84 (s, 1H), 4.67 – 4.63 (m, 1H), 4.53 (d, *J* = 8.8 Hz, 1H), 4.37 – 4.33 (m, 1H), 4.29 – 4.24 (m, 2H), 4.05 – 4.01 (m, 1H), 3.86 (dd, *J* = 12.5, 2.8 Hz, 1H), 3.75 (dd, *J* = 12.4, 3.3 Hz, 1H), 3.66 (d, *J* = 14.0 Hz, 1H), 3.62 (dd, *J* = 10.2, 5.6 Hz, 1H), 3.53 – 3.50 (m, 1H), 2.86 – 2.79 (m, 2H), 2.67-2.34 (m, 9H), 2.59 (s, 3H), 2.56 (s, 3H), 2.28 (s, 3H), 2.29 (s, 3H), 2.19-1.80 (m, 10H) 1.86 (s, 3H), 1.70 – 1.65 (m, 1H), 1.42 (s, 3H), 1.39 (s, 6H), 1.25 (d, *J* = 6.2 Hz, 3H), 1.18 (s, 3H), 1.16-1.10 (m, 2H), 0.45 (s, 3H).

^{13}C NMR (150 MHz, CD_3OD) δ 181.5, 180.3, 178.0, 177.8, 177.3, 176.6, 175.5, 175.3, 174.62, 174.58, 174.2, 166.9, 165.4, 144.0, 138.5, 135.7, 133.9, 131.5, 117.7, 112.5, 109.7, 106.2, 94.9, 87.6, 87.0, 83.4, 76.5, 75.2, 73.6, 70.8, 62.1, 60.6, 57.3, 56.3, 54.3, 52.7, 49.9, 48.3, 46.8, 44.3, 43.1, 40.0, 36.2, 33.4, 32.9, 32.8, 32.7, 32.2, 31.4, 20.8, 20.6, 20.3, 20.2, 20.15, 20.13, 19.9, 17.6, 17.1, 16.2, 15.8.

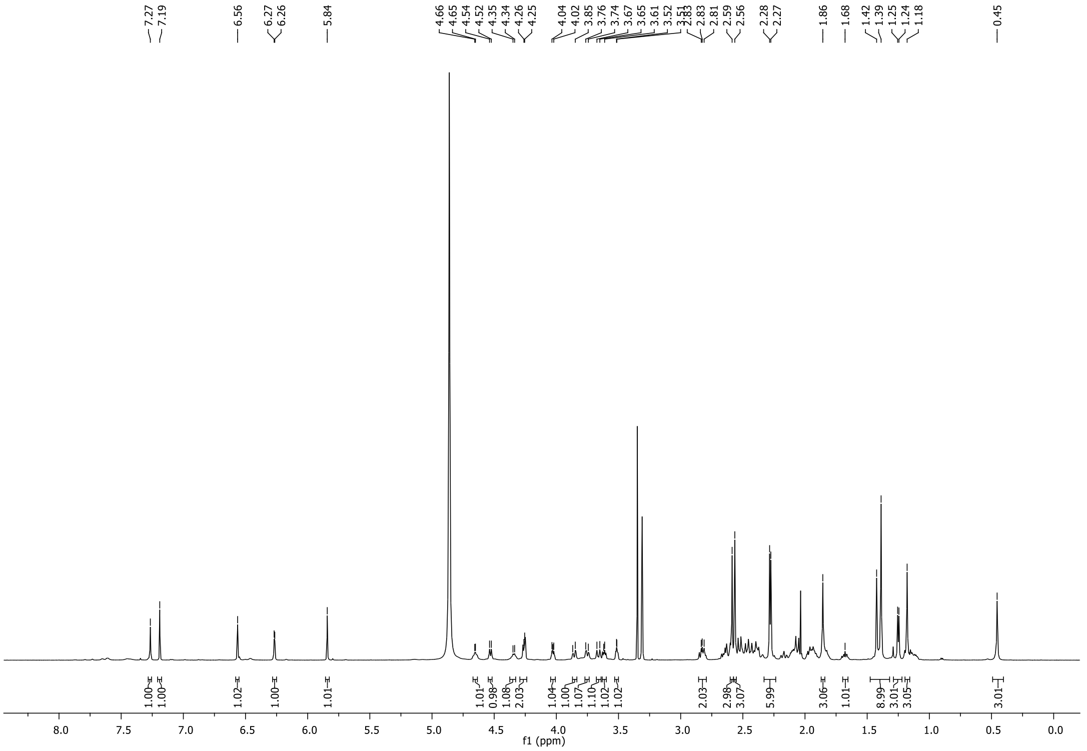

Figure S26. ^{1}H NMR spectrum of (CN)13-*epi*-Cbl (solvent: CD_3OD).

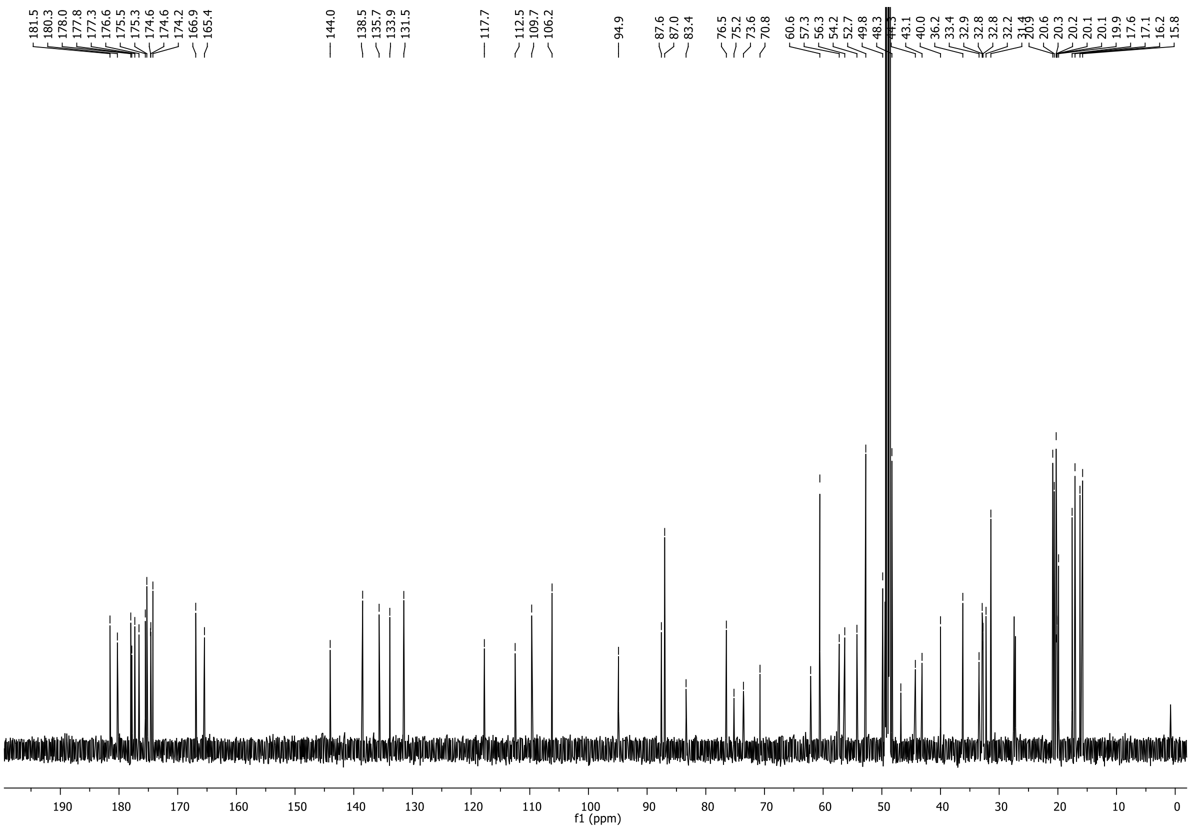

Figure S27. ^{13}C NMR spectrum of (CN)13-*epi*-Cbl (solvent: CD_3OD).

References

[1] M. D. Hanwell, D. E. Curtis, D. C. Lonie, T. Vandermeersch, E. Zurek, G. R. Hutchison, *J Cheminform* **2012**, *4*, 17.

[2] A. R. Allouche, *J. Comput. Chem.* **2011**, *32*, 174–182.

[3] J. Wang, P. Cieplak, P. A. Kollman, *J. Comput. Chem.* **2000**, *21*, 1049–1074.

[4] Gaussian 16, Revision C.01, M. J. Frisch, G. W. Trucks, H. B. Schlegel, G. E. Scuseria, M. A. Robb, J. R. Cheeseman, G. Scalmani, V. Barone, G. A. Petersson, H. Nakatsuji, X. Li, M. Caricato, A. V. Marenich, J. Bloino, B. G. Janesko, R. Gomperts, B. Mennucci, H. P. Hratchian, J. V. Ortiz, A. F. Izmaylov, J. L. Sonnenberg, D. Williams-Young, F. Ding, F. Lipparini, F. Egidi, J. Goings, B. Peng, A. Petrone, T. Henderson, D. Ranasinghe, V. G. Zakrzewski, J. Gao, N. Rega, G. Zheng, W. Liang, M. Hada, M. Ehara, K. Toyota, R. Fukuda, J. Hasegawa, M. Ishida, T. Nakajima, Y. Honda, O. Kitao, H. Nakai, T. Vreven, K. Throssell, J. A. Montgomery, Jr., J. E. Peralta, F. Ogliaro, M. J. Bearpark, J. J. Heyd, E. N. Brothers, K. N. Kudin, V. N. Staroverov, T. A. Keith, R. Kobayashi, J. Normand, K. Raghavachari, A. P. Rendell, J. C. Burant, S. S. Iyengar, J. Tomasi, M. Cossi, J. M. Millam, M. Klene, C. Adamo, R. Cammi, J. W. Ochterski, R. L. Martin, K. Morokuma, O. Farkas, J. B. Foresman, and D. J. Fox, Gaussian, Inc., Wallingford CT, **2019**.

[5] R. D. Dennington II, T. A. Keith, J. M. Millam, GaussView 6.0.16, Copyright, Semichem, Inc. **2000-2016**.

[6] W. Humphrey, A. Dalke, K. Schulten, *J. Mol. Graph.* **1996**, *14*, 33–38.

[7] N. M. O'boyle, A. L. Tenderholt, K. M. Langner, *J. Comput. Chem.* **2008**, *29*, 839–845.

[8] V. Barone, M. Cossi, *J. Phys. Chem. A* **1998**, *102*, 1995–2001.

[9] T. Wu, G. Li, J. Kapitán, J. Kessler, Y. Xu, P. Bouř, *Angew. Chem. Int. Ed.* **2020**, *59*, 21895–21898.

[10] R. Bonnett, A. Neuberger, G. W. Kenner, *Phil. Trans. R. Soc. Lond. B* **1976**, *273*, 295–301.

[11] R. Bonnett, J. M. Godfrey, V. B. Math, *J Chem Soc C Org* **1971**, 3736–3743.

[12] A. J. Wierzba, A. Wincenciuk, M. Karczewski, V. I. Vullev, D. Gryko, *Chem. Eur. J.* **2018**, *24*, 10344–10356.

[13] K. ó Proinsias, M. Karczewski, A. Zieleniewska, D. Gryko, *J. Org. Chem.* **2014**, *79*, 7752–7757.
